# Developmental trajectories of glutamate and the variable clinical course of ADHD in youth

**DOI:** 10.1038/s41398-026-03898-7

**Published:** 2026-02-13

**Authors:** Marine Bouyssi-Kobar, Yan Zhang, Luke Norman, Saadia Choudhury, Wendy Sharp, Gustavo Sudre, Tonya White, Philip Shaw

**Affiliations:** 1 Section on Social and Cognitive Developmental Neuroscience, National Institute of Mental Health, 10 Center Drive, Bethesda, MD 20892 USA; 2 Magnetic Resonance Spectroscopy Section, National Institute of Mental Health, 10 Center Drive, Bethesda, MD 20892 USA; 3 King’s Maudsley Partnership for Children and Young People, Institute of Psychiatry, Psychology & Neuroscience at King’s College, London, WC2R 2LS United Kingdom

**Keywords:** ADHD, Diagnostic markers, Molecular neuroscience

## Abstract

Recent evidence suggests the brain’s major excitatory neurotransmitter, glutamate, plays a key role in attention-deficit/hyperactivity disorder (ADHD). Here we ask if glutamate also plays a role in the variable clinical courses of ADHD. While some children ‘grow out’ of ADHD by adolescence, others experience persistent symptoms into adulthood. Prior work implicates structural and functional differences in medial prefrontal cortex as pivotal in these different ADHD symptom courses, and we now ask if glutamate developmental change also contributes. Given the role of glutamate in neurotransmission, we also investigate potential impacts on the brain’s intrinsic connectivity. Using a glutamate-specific magnetic resonance spectroscopy sequence at 3 T, we analyzed 241 spectra on 161 participants, including 69 with persistent ADHD, 20 with remitting ADHD, and 72 never affected controls. Intrinsic functional connectivity was also assessed in a subset of 104 participants with 141 functional MRI scans. Using linear mixed models, we found an age-related increase in medial prefrontal glutamate in the persistent ADHD group which differed significantly from an age-related decrease among those who remitted and the never affected controls. Furthermore, altered prefrontal glutamate concentrations were associated with changes in intrinsic connectivity between the default mode network (which includes medial frontal cortex) and subcortical regions. These findings may indicate altered maturation of glutamate in the medial prefrontal cortex in youth with persistent ADHD.

## Introduction

Attention-Deficit/Hyperactivity Disorder (ADHD) is a common neurodevelopmental condition characterized by developmentally inappropriate hyperactivity-impulsivity and/or inattention symptoms [1]. Although dysregulation of the catecholaminergic system (dopamine and noradrenaline) has long been implicated in ADHD [2], several lines of evidence also point to a role for glutamate, the brain’s primary excitatory transmitter [3, 4]. Genome-wide studies have found enrichment of both rare copy number variants and common risk alleles in glutamatergic receptor gene networks [5, 6]. A medication (fasoracetam) that modulates glutamate receptor activity has been found to reduce ADHD symptoms in young people with copy number variants clustering in the metabotropic glutamatergic receptor gene network [7]. Furthermore, post-mortem brain studies in those with lifetime histories of ADHD point toward altered expression of glutamatergic genes in fronto-striatal regions [8].

Glutamate can be directly measured in vivo non-invasively in humans using proton Magnetic Resonance Spectroscopy (MRS). Proton MRS quantifies neurochemicals in focal brain regions (voxels), without the use of contrast agents or ionizing materials, by leveraging differences in the signal produced by hydrogen depending on its binding with biochemicals. MRS findings concerning alteration of glutamate concentration in those with ADHD have been inconclusive, due partly to a reliance on cross-sectional designs, heterogeneity of participants’ age, and technical challenges namely, the use of low-strength magnetic fields and single echo-time spectra, which cannot effectively differentiate glutamate from glutamine, resulting in the reporting of the combined ‘Glx’ MRS signal [9, 10]. In this context, the field can benefit from longitudinal studies that examine the neurotransmitter glutamate using improved techniques capable of mitigating the confounding influence of glutamine [4].

Here, we focus on glutamate levels measured in the medial prefrontal cortex in youth with ADHD that have either persisted or remitted from early childhood. We focus on the medial prefrontal cortex for three reasons. First, the medial prefrontal cortex mediates cognitive processes implicated in ADHD, including the allocation of executive attention [11], decision making [12, 13], and emotional regulation [14, 15]. Secondly, systematic reviews have pointed to altered glutamate in the medial prefrontal cortex as one of the more consistent findings in ADHD [9, 10]. Finally, the glutamatergic circuitry of the prefrontal cortex interacts with catecholaminergic systems that also play a role in ADHD symptom formation [16, 17].

It is well established that childhood ADHD follows highly variable courses into adolescence: while some children show ADHD symptoms that persist into adolescence, others show marked improvement, retaining variable degrees of diagnostic remission [18]. In a study using longitudinal structural neuroimaging, we found that children whose ADHD symptoms resolve by late adolescence also show a trajectory of convergence towards typical dimensions in the thickness of the medial prefrontal cortex [19]. By contrast, children with persistent ADHD follow an ‘atypical’ neurodevelopmental trajectory. We now ask if a similar principle holds for the trajectories of glutamate in the medial prefrontal cortex. Specifically, we hypothesize that children with persistent ADHD will show an atypical glutamate trajectory, whereas those who have remitting ADHD will show a more typical developmental trajectory of glutamate levels in the medial prefrontal cortex.

Finally, we explore possible associations between altered glutamate in ADHD and altered functional connectivity. We do so as ADHD has been conceptualized as resulting from atypical interactions between brain networks, particularly a loss of the usual counterbalance between the default mode network (‘task free’) and task positive networks such as those of attention and cognitive control [20, 21]. There is evidence that glutamate may play a role in the regulation of functional connectivity as measured by functional magnetic resonance imaging [22–24]. Thus, altered glutamate in those with ADHD may also be tied to altered interaction between the brain’s functional networks. To explore this possibility, we use MRS and resting-state functional MRI (rs-fMRI) data acquired on the same subjects, to determine if altered glutamate concentration in our medial prefrontal cortex region of interest, which is part of the brain’s default mode network, is also associated with differences in the connectivity of the default mode network.

## Materials and methods

### Ethics approval and consent to participate

We selected participants with glutamate MRS data from an accelerated longitudinal cohort study, the Neurobehavioral Clinical Research (NCR) study (ClinicalTrials.gov ID: NCT01721720) [25]. The study was approved by the Institutional review board of the National Institutes of Health (FWA00005897). Informed consent was obtained from all participants: parents and adults gave informed, written consent and the youth gave written or verbal assent. All methods were performed in accordance with the relevant guidelines and regulations.

### Participants

The NCR cohort is enriched for those who met diagnostic criteria for ADHD. ADHD symptoms were evaluated by experienced clinicians using the parental Diagnostic Interview for Children and Adolescents-IV [26] and the Structured Clinical Interview for DSM-5 [27] for participants 18 years-of-age and above. The MRS data were collected during the final years of a larger longitudinal study, and we used all available clinical data to define diagnostic trajectories. Persistent ADHD was defined as participants who met diagnostic criteria for ADHD during the study period and still met criteria at final follow-up. Remitting ADHD met criteria at the time of study entry but not at a later observation. Our control group consists of typically developing youth free of psychiatric diagnosis and is referred to as the “never affected” group. The clinical observations and timepoints during which the individual also had MRS estimates of glutamate are given in Supplementary Fig. 1. Principal exclusion criteria were a full-scale intelligence quotient (IQ) < 80, evidence of neurological disorders known to affect brain structure, current substance dependence, psychotic disorders, and medications that target glutamate. Full-scale IQ was assessed using age-appropriate versions of the Wechsler intelligence scale [28, 29]. Medication history was ascertained and participants receiving psychostimulant medication (methylphenidate-based or amphetamine-based prescription drug) withheld for 24 h prior to imaging assessments.

### Data acquisition

**Fig. 1:**
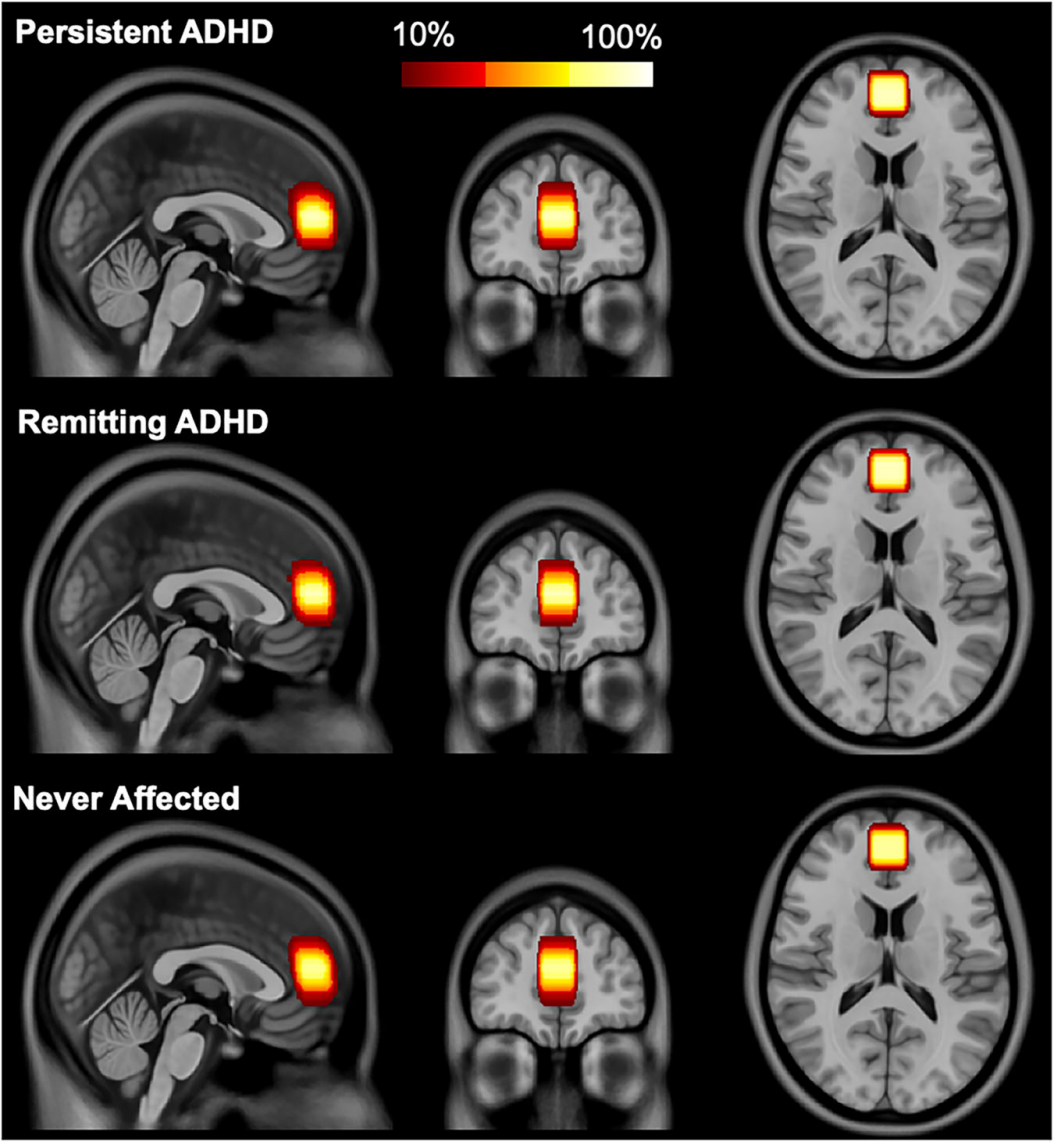
Region of interest in medial prefrontal cortex for Magnetic Resonance Spectroscopy (MRS) by outcome group. Voxel spatial percent overlap across all participants from the same outcome group represented in the Montreal Neurological Institute (MNI) space using the ICBM 2009c Nonlinear Asymmetric template available at https://nist.mni.mcgill.ca/icbm-152-nonlinear-atlases-2009.

Magnetic resonance data were acquired on a GE 3 Tesla Discovery MR750 system with an 8-channel head coil (General Electric Medical Systems, Milwaukee, WI); the same unit was used throughout the study period, and no major hardware or software upgrades occurred. We used a dedicated glutamate J-point resolved spectroscopy (JPRESS) sequence that allows for glutamate and glutamine simultaneous quantification [30] to quantify glutamate within the medial prefrontal cortex (Fig. 1). Detailed parameters are provided according to the current MRS guidelines for reporting acquisitions parameters and quality metrics [31] in the Supplementary materials (Supplementary Table 1, Supplementary Fig. 2) and described briefly here: resolution: 2x2x2 cm^3^; repetition time: 2000 ms; 32 echo times ranging from 35 ms to 221 ms with a step size of 6 ms; number of averages: 4; 4096 sampling points for each echo; 5 kHz spectral width; and scan time: 5.3 min. A single midline MRS voxel was positioned in the medial prefrontal cortex at the level of the anterior cingulate, guided by the T1-weighted image. Placement was determined from the mid-sagittal slice, where the bottom of the voxel was aligned just anterior to the genu of the corpus callosum, and then adjusted in the axial plane to maximize coverage of bilateral gray matter. During the same scanning session, two consecutive resting-state fMRI runs were acquired back-to-back, each lasting 5 min for a total of 10 min, while participants viewed a fixation cross. Data were collected with axial gradient echo planar imaging at 3.4 × 3.4 × 2.8 mm^3^ resolution, repetition time: 2500 ms, echo time: 27 ms, flip angle: 90°, field of view: 22 cm, acquisition matrix: 64×64, 120 volumes per run. The high-resolution anatomical T1-weighted image had the following parameters: magnetization prepared rapid acquisition gradient recalled echo sequence (MP-RAGE); 1x1x1 mm^3^ voxel; repetition time: 8 ms, echo time: 3 ms; inversion time: 900 ms; flip angle: 7°; field of view: 22 cm; acquisition matrix: 256×256; acquisition time: 5.3 min.

### Data processing

#### Proton magnetic resonance spectroscopy data

MRS quantification of metabolites was performed using a custom-developed, fully automated program [30, 32]. The 32 spectra acquired at different echo times were modulated using spin coupling constants of J = 0 and J = 7.5 Hz, respectively, resulting in two spectra (Supplementary Fig. 2). These were concatenated into a single 1D spectrum and subsequently fitted using basis sets generated via quantum mechanical simulation, as described in Zhang and Shen (2016). The spectra were scaled using a corrected water reference, with the contribution of cerebrospinal fluid (CSF) removed [30]. Metabolite concentration was corrected for partial volume effects following the segmentation of the T1w image using Freesurfer (v7.4.1) [https://surfer.nmr.mgh.harvard.edu/; [33]] and computation of the gray matter and white matter voxel composition (details in Supplementary Table 1). Exclusion criteria for MRS spectrum were signal- to-noise ratio < 5 (measured on the peak of N-acetyl aspartate), full width at half maximum > 0.1 ppm, concentration of metabolites outside three standard deviations of the mean, and wrong voxel placement (e.g., not bilateral) or inability to extract voxel composition (e.g., anatomical sequence corrupted by motion).

#### Resting-state functional MRI (rs-fMRI) data

Functional MRI is based on the blood oxygen level dependent signal and

rs-fMRI data processing is described elsewhere [34]. In summary, we used a validated 36-parameter plus despiking preprocessing pipeline [35–37] using the fMRIPrep and eXtensible Connectivity Pipeline (XCP) software [38, 39]. Variations in blood oxygen level dependent MRI signal (time course) were extracted by regions of interest (ROIs) from the Schaefer atlas for cortical networks [40] mapped to Yeo’s seven-network parcellation [41] and previously defined subcortical ROIs [20] including caudate, putamen (dorsal and ventral), thalamus, ventral striatum, and amygdala (dorsal and ventral). Given that subcortical subdivisions share substantial variance in their time series [20], we initially combined these regions for our primary analysis and subsequently performed exploratory post-hoc analysis using the aforementioned subcortical ROIs. Correlations between the time course of each ROI were computed for each participant followed by a Fisher’s r-to-z transformation resulting in an ROI-to-ROI connectivity matrix for each participant. Given that (1) the position of our MRS voxel is located mainly in the default mode network (DMN) and (2) DMN alterations have been shown in ADHD [42, 43], we selected a-priori network metrics related to the DMN. These metrics were average connectivity between nodes of the DMN (“within DMN connectivity”) as well as connectivity between the DMN and the dorsal attention, salience, central executive, and subcortical networks. Within DMN connectivity was calculated by averaging all the pairwise combinations of ROIs belonging to the DMN, and between network DMN connectivity was computed by averaging all the pairwise combinations of DMN ROIs to the ROIs of the other networks of interest [42]. We excluded rs-fMRI data with excessive motion (mean framewise displacement > 0.3 mm) and only included scans with two usable runs (i.e., 10 min of rs-fMRI data).

### Statistical analyses

Statistical analyses were performed in R (version 4.4.0 [44]) using the following packages: *stats* [44] (v4.4.0), *nlme* [45, 46] (v3.1.167), *lmeresampler* [47, 48] (v. 0.2.4), *report* [49] (v0.6.1), and *effectsize* [50] (v0.8.9). Normality was assessed using the Shapiro-Wilk normality test. To determine the optimal model for age-related changes, we compared linear, quadratic, and cubic polynomial models using the Akaike information criterion (AIC). The linear model provided the best fit across all groups (Supplementary Table 2), supporting the use of a linear age term in subsequent analyses.

The mixed model used was:

*Glutamate*_*ij*_ *~ outcome_group*_*i*_ * age_*ij*_ *+ sex*_*i*_ *+ glutamate_CRLB*_*ij*_, with a random intercept term for subject ( ~ 1|ID).

Glutamate is the corrected glutamate concentration in millimolar (mM) for individual i at time j, outcome_group is persistent ADHD, remitting ADHD, or never affected, and glutamate_CRLB is the Cramér Rao Lower Bounds (CRLB) of glutamate. Bootstrapped 95% confidence interval of the beta coefficient estimate are given (Parametric bootstrap with replacement, 1000 resamples) as well as standardized beta coefficient. To examine the significance of the fixed effects, of the linear mixed model, first we applied an ANOVA to the linear model. For thoroughness and comparison with other studies, we also computed statistics with other metabolites including glutamine and Glx (glutamine + glutamate) as well as total N-acetyl aspartate, total Creatine, total Choline and Gamma-aminobutyric acid (GABA).

Additionally, we tested whether the association between glutamate and network metric differs by outcome group through the following model:

*Network_metric*_*ij*_ *~ Glutamate*_*ij*_ * outcome_group_*i*_ *+ age*_*ij*_ *+ sex*_*i*_ *+ motion*_*ij*_ + *(motion*_*ij*_*)*^*2*^ ; with a random intercept term for subject (~ 1|ID).

Network_metric is one of the following measures described in the rs-fMRI processing method section: within DMN connectivity, and connectivity between DMN-dorsal attention network, DMN-salience network, DMN-central executive network, and DMN-subcortical network. Due to the sparsity of resting-state fMRI longitudinal data, we did not consider interaction with age; rather we accounted for age in our model. To correct for multiple comparisons of the five-network metrics of interest of our primary analysis, the significance was set to p = 0.05/5 = 0.01. Subcortical ROIs post-hoc analyses were exploratory in nature, thus we did not apply multiple comparison correction.

#### Sensitivity analyses and robustness checks

Despite group differences in IQ (Table 1), we did not include IQ as a covariate in our primary analyses. This decision was based on the rationale that IQ differences may be intrinsic to the ADHD phenotype rather than a confounding factor, and that covarying IQ could inadvertently remove meaningful variance associated with the condition itself [51, 52]. Nonetheless, to further evaluate the robustness of our results, in sensitivity analyses we covaried for IQ.

**Table 1:** Clinical characteristics of the cohort.

	Persistent ADHD (N = 69)	Remitting ADHD (N = 20)	Never Affected (N = 72)	Statistics
**Sex (Female (F);Male (M))**	14 F (20%) 55 M (80%)	5 F (25%) 15 M (75%)	26 F (36%) 46 M (64%)	χ^2^_(2)_ = 4.478 p-value = 0.11
**Race**				
American Indian or Alaska Native	1	0	0	χ^2^_(8)_ = 9.884 p-value = 0.27
Asian	0	0	5	
Black or African American	12	3	7	
Mixed	7	3	11	
White	49	14	49	
**Ethnicity**				
Hispanic or Latino	9	3	5	χ^2^_(4)_ = 3.264 p-value = 0.51
Not Hispanic or Latino	59	17	67	
Unknown	1	0	0	
**Age at baseline MRS scan (years)** mean ± SD	13.86 ± 2.99	15.07 ± 2.46	13.75 ± 3.07	F_(2,158)_ = 1.603 p-value = 0.2
**Follow-up MRS scan performed**, N (%)	29 (42%)	9 (45%)	34 (47%)	χ^2^_(2)_ = 0.385 p-value = 0.82
**Age at follow-up MRS scan (years)** mean ± SD	15.49 ± 3.59	15.86 ± 2.37	14.97 ± 2.68	F_(2,69)_ = 0.4 p-value = 0.67
**Time interval between baseline and follow-up scans (years)**, mean ± SD	2.16 ± 0.83	2 ± 0.7	1.9 ± 0.69	F_(2,69)_ = 0.63 p-value = 0.53
**Age at first clinical observation (years),** mean ± SD	9.01 ± 2.73	9.42 ± 2.41	9.45 ± 2.8	F_(2,158)_ = 0.49 p-value = 0.61
**Inattentive symptoms, at first clinical observation,** median [range]	6[0;9]	6 [1;9]	0 [0;5]	K-Wχ^2^_(2)_ = 104.67 p-value < 2.2 ×10^−16^
**Inattentive symptoms, at last clinical observation,** median [range]	6[1;9]	3[0,5]	0 [0;5]	K-Wχ^2^_(2)_ = 114.2 p-value < 2.2 × 10^−16^
**Hyperactive-Impulsive symptoms at first clinical observation**, median [range]	5 [0;9]	3.5[0,9]	0 [0;4]	K-Wχ^2^_(2)_ = 87.323 p-value < 2.2 × 10^−16^
**Hyperactive-Impulsive symptoms at last clinical observation**, median [range]	3 [0;9]	1.5 [0;5]	0 [0;4]	K-Wχ^2^_(2)_ = 64.056 p-value < 1.23 × 10^14^
**Psychostimulant medication at any clinical observation**, n (%)	48 (70%)	11 (55%)	0	*Persistent vs. Remitting ADHD:* χ^2^_(1)_ = 0.89 p-value = 0.34
**Psychostimulant medication at first MRS observation**, n (%)	44 (64%)	6 (30%)	0	*Persistent vs. Remitting ADHD:* χ^2^_(1)_ = 5.88 p-value = 0.02
**Psychostimulant medication at last clinical observation**, n (%)	33 (48%)	6 (30%)	0	*Persistent vs. Remitting ADHD:* χ^2^_(1)_ = 1.34 p-value = 0.25
**Other Diagnosis**^a^, n (%)	13 (19%)	0	0	*Persistent vs. Remitting ADHD:* χ^2^_(1)_ = 3.03 p-value = 0.08
**Full scale IQ**	106.16 ± 13.59	110.4 ± 11.6	112.95 ± 13.56	F_(2,158)_ = 4.595 p-value = 0.01

*IQ*, intelligence quotient; *MRS*, magnetic resonance spectroscopy.

^a^Other Diagnosis includes social anxiety (n = 2), generalized anxiety disorder (n = 2), obsessive-compulsive disorder (n = 1), disruptive mood dysregulation (n = 1), and oppositional defiant disorder (n = 7).

In addition, although all participants underwent a 24-hour washout period, we conducted Supplementary analyses controlling for both psychostimulant status at the time of scan and any psychostimulant medication use during the study period. This approach was based on prior literature suggesting that psychostimulant medication may influence medial prefrontal glutamate levels [53–55].

The majority of ADHD participants were free of psychiatric comorbidities (76 out of 89); the 13 participants with comorbidities were all in the ADHD persistent group. Nonetheless, given evidence that conditions such as anxiety and depression have been linked to altered glutamate, we repeated the analyses after excluding the 13 participants with persistent ADHD who met criteria for comorbid disorders.

Finally, we examined potential sex-specific effects by testing for three-way interactions between age, outcome group, and sex. This analysis was conducted to determine whether the age-related trajectories of glutamate levels across the three outcome groups (persistent ADHD, remitting ADHD, and never affected) differed between males and females.

## Results

### Demographics and clinical data

We acquired 283 MRS data on 175 participants, who had a total of 815 clinical observations (Supplementary Fig. 1). Following quality control, we retained 241 spectra (Supplementary Fig. 3) from 161 participants (Supplementary Fig. 4 shows reasons for exclusion). Of these 161 subjects, 69 had persistent ADHD, 20 remitting ADHD and 72 were never affected (Table 1). Sex at birth, race/ethnicity, age at baseline scan, and number of MRS spectra and their quality metrics did not differ significantly between groups (Table 1 and Supplementary Table 3). The outcome groups also did not differ in the interval between baseline and follow-up scans (F_(2,69)_ = 0.63, p = 0.53): persistent ADHD (N = 69; 29 with follow-up scans): 2.16 ± 0.83 years; remitting ADHD (N = 20; 9 with follow-up scans): 2.00 ± 0.70 years; and never affected (N = 72; 34 with follow-up scans): 1.90 ± 0.69 years.

### Group differences in the developmental change in glutamate

**Fig. 2:**
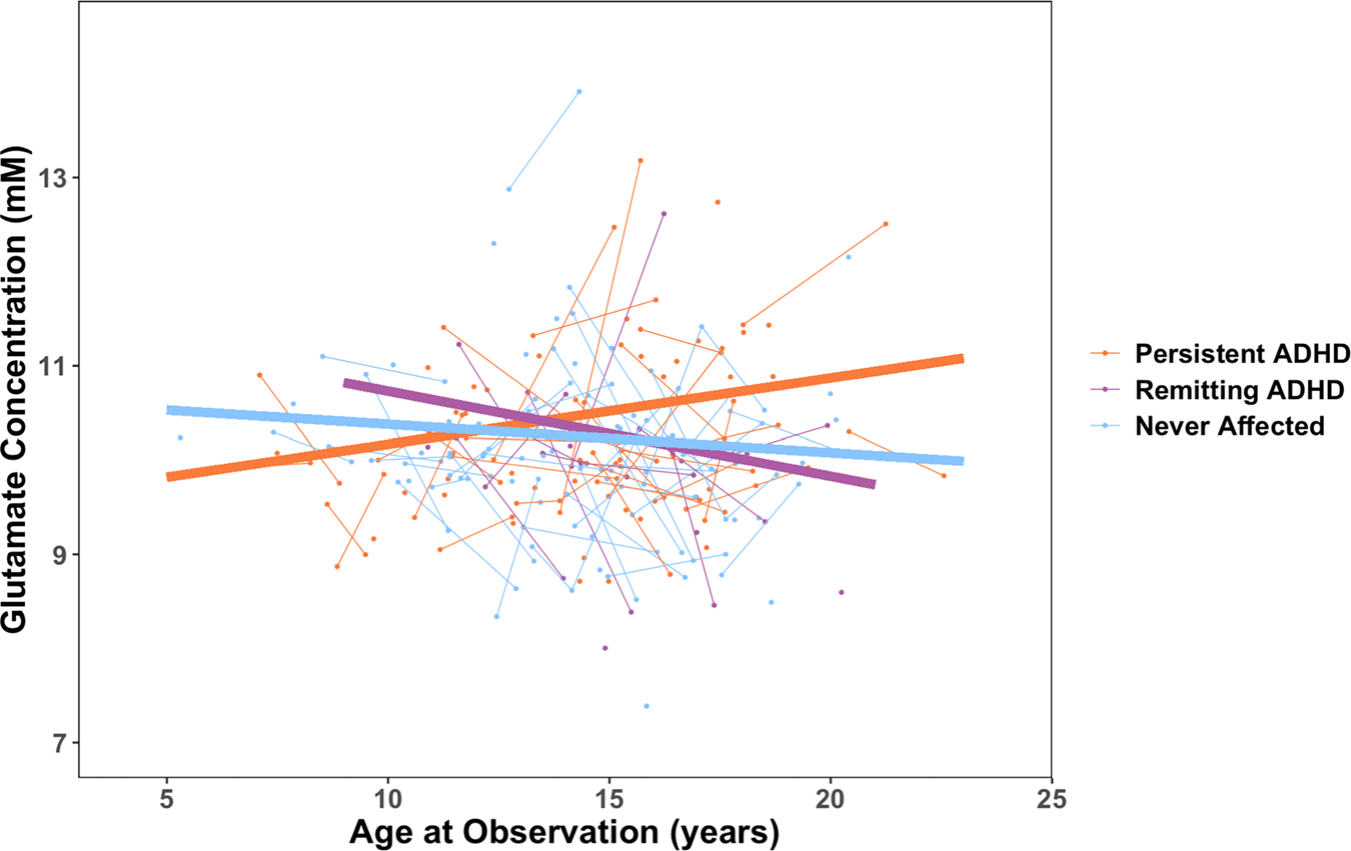
Developmental Trajectory of Glutamate by Outcome Group. Points and thin lines represent individual participant data, and thick lines are the outcome group fitted slope from the following mixed model: *Glutamate*_*ij*_ *~ outcome_group*_*i*_ * age_*ij*_ *+ sex*_*i*_ *+ glutamate_CRLB*_*ij*_, with a random intercept term for subject (~1|ID). Slope (β estimate) by outcome group were as follows: persistent ADHD: 0.07 mM/year; remitting ADHD: −0.09 mM/year; never affected: −0.03 mM/year. Glutamate trajectory in persistent ADHD was significantly different from remitting group (t_(76)_ = 2.18, p = 0.032) and never affected group (t_(76)_ = 2.28, p = 0.026), whereas remitting group glutamate trajectory was not statistically different from the never affected group (t_(76)_ = −0.94, p = 0.35).

Glutamate concentrations at the time of the first scan did not differ by diagnosis (ADHD at any time point versus never affected) nor by outcome groups (persistent ADHD, remitting ADHD, never affected) – (Supplementary Table 4). We did find a significant difference between the three outcome groups in the rate of change in absolute glutamate concentration (F_(2,76)_ = 3.89, p = 0.02). The persistent ADHD group showed an age-related increase in glutamate, compared to a decrease shown by the never affected group (persistent ADHD*Age: beta = 0.096, 95% bootstrap CI [0.01, 0.18], Standardized (Std.) beta = 0.32, 95% CI [0.04, 0.60], t_(76)_ = 2.28, p = 0.026; Fig. 2. The remitting ADHD group also showed an age-related decrease in glutamate that differed from the persistent ADHD group (remitting ADHD*Age: beta = −0.17, 95% bootstrap CI [−0.3, −0.025], Std. beta = −0.57, 95% CI [−1.08, −0.05], t_(76)_ = -2.18, p = 0.032) but not the never affected group (remitting ADHD*Age: beta = −0.07, 95% bootstrap CI [−0.22, 0.09], Std. beta = −0.24, 95% CI [−0.76, 0.27], t_(76)_ = −0.94, p = 0.35). Neither glutamine concentration nor the ratio of glutamate/total creatine showed any differences in development between outcome groups (Supplementary Table 5). Following robustness analyses, our main findings remain when controlling for IQ, psychostimulant medication at neuroimaging or ever on psychostimulant medication, and when removing participants with comorbidities (Supplementary Table 6.A). Furthermore, the three-way interaction between age, outcome group, and sex was not significant (p = 0.39; model including persistent ADHD vs. never affected only due to lack of convergence of the full model; Supplementary Table 6.B), indicating that age-related glutamate trajectories across outcome groups did not differ by sex. Finally, when removing the remitting group, the results also showed that the persistent ADHD group glutamate trajectory was significantly different from the never affected group (Supplementary Table 6.C).

While we had no specific hypotheses concerning other metabolites, total N-acetyl aspartate trajectories also differed by outcome groups (F_(2,76)_ = 3.91, p = 0.02) with the persistent ADHD group exhibiting a steeper increase with age compared to the never affected group (persistent ADHD*Age: beta = 0.07, 95% bootstrap CI [0.01, 0.13], Std. beta = 0.32, 95% CI [0.04, 0.6], t_(76)_ = 2.36, p = 0.021; see also Supplementary Table 7). Other major metabolites including total creatine, total choline, and GABA did not show outcome group differences (Supplementary Table 7). Of note, the MRS sequence and quantification method used were not specific to GABA quantification, thus GABA is only mentioned for reporting purposes.

### Relationship between medial prefrontal glutamate and rs-fMRI brain connectivity

After quality control, 104 participants with 141 observations had both rs-fMRI data and MRS data (35 participants had more than one observation). Of these 104, 39 had persistent ADHD, 16 had remitting ADHD and 49 were never affected. Given the very small sample of remitting ADHD (out of the 16 participants with MRS and rs-fMRI data, only 3 had repeated observations), we did not include this group in our main resting-state analysis. The relationship between glutamate concentration and connectivity between the DMN and subcortical network differed significantly between the persistent ADHD group and the never affected group (Std. beta = 0.54, 95% CI [0.14, 0.94], t_(29)_ = 2.78, p = 0.009). Additionally, there was a nominally significant group difference in the relationship between glutamate concentration and both connectivity within the DMN (Std. beta = 0.5, 95% CI [0.1, 0.91], t_(29)_ = 2.53, p = 0.017) and connectivity between the DMN and central executive network (Std. beta = 0.41, 95% CI [0.04, 0.78], t_(29)_ = 2.28, p = 0.03). Higher glutamate concentration in the medial prefrontal cortex in the persistent ADHD group was associated with increased connectivity within the DMN and increased connectivity between the DMN and subcortical networks and DMN and central executive network (Supplementary Table 8.A and Supplementary Fig. 5.A). Findings remain when controlling for IQ, psychostimulant medication, and comorbidities (Supplementary Table 8.A). Of note, the model still converges when we include the remitting group, and results remain similar with the significant differences being only between the persistent ADHD vs. never affected group (Supplementary Table 8.C).

Although we initially treated subcortical regions as a combined network by averaging across regions, we recognize that subcortical structures do not form functional networks in the same way as cortical regions. Following significant findings in the combined subcortical analysis, we conducted exploratory post-hoc analyses to identify which specific subcortical regions drove this effect. These analyses revealed that the caudate and dorsal amygdala showed significant group differences in the relationship between glutamate levels and connectivity with the DMN (Supplementary Table 8.B and Supplementary Fig. 5.B), while other subcortical regions did not show significant difference.

## Discussion

To our knowledge, this is the first study to examine how developmental change in glutamate may track with the course of ADHD from childhood into adolescence. We find that youth with persistent ADHD show an age-related increase in glutamate levels, whereas youth whose ADHD remitted show a more typical pattern of glutamatergic development in the medial prefrontal cortex. We also find those with persistent ADHD have an atypical relationship between glutamate concentration and intrinsic connectivity both within the DMN and between the DMN and subcortical network. An overall pattern emerges of subtle developmental alterations in brain maturation in persistent ADHD, while remitting ADHD youth exhibit neural features that more closely resemble those of never affected individuals.

Previous studies of medial prefrontal glutamate development, measured by MRS, find an increase during early childhood [56–58], followed by an age-related decrease into adolescence and adulthood [59–62]. Our data from never affected control individuals also show a decrease in glutamate from late childhood into adolescence, although we lack sufficient numbers of young children to capture the likely phase of glutamate increase. One interpretation of the age-related increase in glutamate that we find in the persistent ADHD group is that this represents the possibility of delayed neurodevelopment. If this hypothesis is true, with continued follow-up we would eventually capture a phase of decrease in glutamate levels. Alternatively, individuals whose ADHD persists into adulthood may maintain elevated glutamate levels throughout development. In this case, the elevated glutamate tracks with the disorder more so than with development. Parsing these scenarios requires longer longitudinal studies with multiple follow-ups.

These results add to growing evidence for a role for glutamate in ADHD pathophysiology [3] complementing evidence from genomics [5, 6], transcriptomics [8], and pharmacology [7, 63]. Interestingly, the glutamate differences only emerged when we assessed trajectories through a developmental lens and were able to consider the highly dynamic nature of ADHD itself. This highlights the advantages of using longitudinal designs to characterize developmental processes that are central to ADHD. The age-related glutamate differences we observed might also help explain the inconsistent results from cross-sectional studies [53, 64–69], as it becomes clear that studies conducted in early childhood may yield very different results from those recruiting at later ages. However, not all participants contributed longitudinal clinical observations following their MRS scans, limiting our ability to assess whether glutamate trajectories could serve as prognostic indicators. The results should therefore be interpreted as providing mechanistic insights rather than predictive biomarkers, extending accumulating evidence, including our group’s earlier anatomical work [19, 70], that ADHD remission involves normalization of neurodevelopment or compensation while persistence reflects sustained or progressive alterations [71–74].

We found that those with persistent ADHD have an atypical relationship between glutamate and default mode network connectivity [21, 42, 43, 75]. This finding is consistent with findings that glutamate plays a role both in the typical development of the brain’s intrinsic functional architecture [22–24, 76, 77] and evidence that altered glutamate levels are associated with atypical neural connectivity in other psychiatric challenges including psychosis [78] and trauma [79]. The association between glutamate and altered connectivity between DMN and the caudate and dorsal amygdala in those with persistent ADHD is of particular note. Atypical interactions between the caudate and prefrontal regions (including the medial prefrontal cortex which lies within the DMN) in particular have long been implicated in ADHD [20, 67, 68, 80–83], and here we suggest that altered medial prefrontal glutamate may play role.

An additional observation was that youth in the remitting group had relatively higher IQ scores and no psychiatric comorbidities. In contrast, lower IQ (though still within the normal range) and comorbid diagnoses were often present among those with persistent ADHD. These patterns are in line with prior reports that both lower cognitive ability and psychiatric comorbidity can hinder remission and increase the likelihood of persistence [84, 85]. Furthermore, although differences did not reach statistical significance, numerically more of the persistent group were medicated during the study (70% vs. 55% ever medicated) including at final follow-up (48% vs. 30%). These factors could potentially confound our glutamate findings, as cognitive ability has been linked to glutamatergic function [86], psychiatric comorbidities often involve glutamate dysregulation [87, 88], and both acute and long-term psychostimulant medication can modulate glutamate signaling [54]. However, our sensitivity analyses showed that the main group differences in medial prefrontal glutamate trajectories remained unchanged when IQ was included as a covariate, when participants with comorbidities were excluded, and when medication status was controlled for. This indicates that the glutamate findings are not simply driven by these clinical characteristics or treatment effects, but rather reflect neurodevelopmental processes linked specifically to ADHD course. Notably, the fact that the remitting group achieved and maintained symptom remission despite only 30% being on medication at final observation further supports the interpretation that remission reflects genuine developmental neurobiological maturation rather than acute pharmacological effects.

There are several limitations to consider. First, while the study benefits from the use of a glutamate sequence designed for 3 T, it is limited to one predefined brain region: the medial prefrontal cortex. The advent of higher field strengths may augur even more precise quantification of glutamate and the possibility to simultaneously quantify the inhibitory neurotransmitter GABA [89] as well as fast whole brain MRS sequences [90]. Second, although GABA measurement would provide valuable insights into excitation/inhibition balance during adolescent prefrontal development [91], particularly given altered GABA in adult ADHD and associations with impulsivity and inattention [92–94], our MRS sequence was not optimized for GABA quantification. Future studies should employ GABA-edited sequences (e.g., MEGA-PRESS) [95] to examine glutamate-GABA interplay in ADHD trajectories. Third, although the study benefited from longitudinal data and a relatively large sample size for an MRS study, repeated data were not available for all participants, a limitation partly mitigated by the use of linear mixed models, which are designed for the combination of cross-sectional and longitudinal observations in the delineation of trajectories [45]. Fourth, few subjects had repeated resting-state fMRI data, meaning that we could not parse developmental effects of glutamate on default mode network development, but we did show an overall association. Fifth, we did not account for pubertal and hormonal effects. Adolescence involves substantial hormonal fluctuations that influence prefrontal cortical maturation and excitatory-inhibitory balance [96–99]. These processes could affect glutamate concentrations and functional connectivity, but we lacked sufficient pubertal status measures. Finally, it is likely that more subjects would fall within the remitted group if the duration of follow-up was extended, and others may show complicated trajectories of switching between meeting and not meeting diagnostic criteria, so-called ‘fluctuant’ ADHD [100].

In conclusion, our study provides further developmental evidence of altered medial prefrontal glutamate levels in youth with persistent ADHD, underscoring the necessity for longitudinal studies to elucidate the complex interplay between neurodevelopmental disorders and typical brain development. Additionally, our exploratory findings reveal potential links between functional network disturbances characteristic of ADHD and glutamate levels in the brain.

## Supplementary information

Supplementary Materials

## Data Availability

We will share de-identified data where the participant or participant’s parent has given consent for sharing. These data will be available by the end of 2026 on the database of Genotypes and Phenotypes (#55949; dbGaP; https://www.ncbi.nlm.nih.gov/gap/).
