# Supplementary material for: Developmental trajectories of glutamate and the variable clinical course of ADHD in youth
Source: Transl Psychiatry. 2026 Feb 13;16:157. doi: 10.1038/s41398-026-03898-7 (PMC13004872; doi:10.1038/s41398-026-03898-7)
Supplement: Supplementary file 1 — Supplementary Materials [file 41398_2026_3898_MOESM1_ESM.pdf]

Supplementary Materials

Developmental trajectories of glutamate and the variable clinical course of ADHD in youth.

Bouyssi-Kobar *et al.*

Table of Content

Supplementary Methods 2

Figure 1: Longitudinal observations by outcome groups. 2
Table 1: Magnetic Resonance Spectroscopy (MRS) Parameters. 3
Figure 2: Exemplary fitting of J-modulated Sample spectra. 5
Table 2. Model determination: Checking the influence of age on glutamate concentration. 6

Supplementary Results.. 7

Figure 3. Averaged MRS Spectra by Outcome Group and Individual participant's spectra...........7
Figure 4. Flow chart of data included...13
Table 3. Data Quality Control. 14
Table 4. Glutamate at baseline: Cross-sectional Analysis...15
Table 5. Developmental Glutamate, Glutamine and "Glx" Analyses...15
Table 6. Developmental Glutamate: Sensitivity analyses and robustness checks.......................16
Table 7. Relationship between outcome group and MRS metabolites. 18
Table 8. Resting-State Networks, Glutamate, and Outcome group. 19
Figure 5. Glutamate and Default-Mode Network (DMN) Connectivity. 25

References 27

Supplementary Methods

Figure 1: Longitudinal observations by outcome groups.

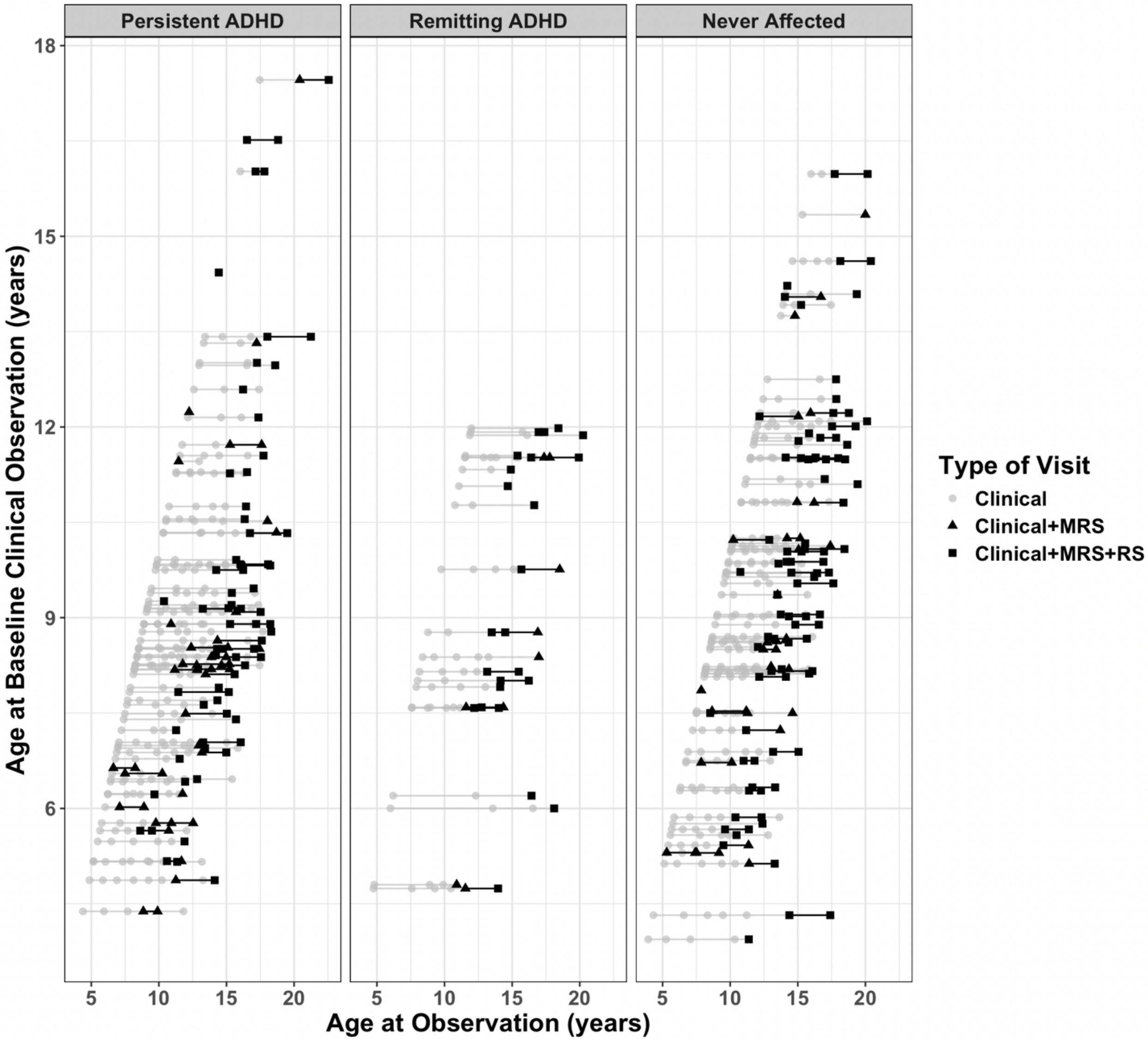

Grey circles represent all the clinical data used, black triangles represent observations for which glutamate magnetic resonance spectroscopy (MRS) was acquired, and black squares the data point where resting-state (RS) fMRI was available alongside MRS.

Table 1: Magnetic Resonance Spectroscopy (MRS) Parameters.

Checklist for minimum reporting standards for MRS [1].

**1. Hardware**	
a. Field strength	3T
b. Manufacturer	General Electric Medical Systems (Milwaukee, Wisconsin, USA)
c. Model (software version if available)	Discovery MR750<br>Software: 27\LX\MR, Software release: DV26
d. RF coils: nuclei (transmit/receive), number of channels, type, body part	^{1}H 8-channel head coil
e. Additional hardware	None
**2. Acquisition**	
a. Pulse sequence	Custom-developed 1D J-point resolved spectroscopy (JPRESS) [2]
b. Volume of interest (VOI) locations	Medial Prefrontal Cortex (see manuscript Figure 2)
c. Nominal VOI size	20 x 20 x 20 mm^3
d. Repetition time (TR), echo time (TE)	TR= 2000 ms<br>TE: from 35 to 221 ms; Echo spacing: 6 ms<br>Total acquisition time: 5.3min
e. Number of echoes, Number of average (NA) per echo	Number of echoes = 32<br>Number of averages = 4
f. Additional sequence parameters	Spectral width: 5 kHz<br>4096 sampling points for each echo
g. Water and fat suppression method	High RF-bandwidth outer volume suppression; automatically fit to voxel boundaries.<br>Two unsuppressed water references were acquired.
h. Shimming method, reference peak, and thresholds for "acceptance of shim" chosen	Manufacturer-supplied prescan for automatic shimming and water frequency lookup.
i. Triggering method	None
j. Frequency and motion correction	None
**3. Data analysis methods and outputs**	
a. Analysis software	Fully automatic spectral fitting program developed in-house [2]; utilizing voxel-based basis spectra generated through full spin density matrix computation [3].
b. Processing steps deviating from quoted reference or product	None.
c. Output measure (e.g., absolute concentration, institutional units, ratio)	Absolute concentration in unit of milli mole (mM)
d. Quantification references and model assumptions	• The JPRESS basis spectra set was generated by spatially localized density matrix simulation [2].<br>• The 32 spectra acquired at different echo times were modulated using spin coupling constants of J = 0 and J = 7.5 Hz, respectively, resulting in two spectra. These were concatenated into a single 1D spectrum and subsequently fitted using basis sets generated via quantum mechanical simulation [2].<br>• The spectra were scaled using a corrected water reference, with the contribution of cerebrospinal fluid removed [2].

	• Anatomical T1 MPRAGE were segmented using (FreeSurfer 7.4.1) [4] to determine the contribution of grey matter (GM) and white matter (WM) within the MRS voxel.<br>• Metabolite concentration C_m was calculated using the following equation [2]:<br><br>$C_m=s_m/S_{ref}(f_{gm}W_{gm}+f_{wm}W_{wm})$<br><br>$s_m$ = fitted metabolite intensity<br>S_{ref} = water reference signal amplitude with CSF removed<br>f_{gm} = volume fraction of GM<br>f_{wm} = volume fraction of WM<br>W_{gm} = pure GM concentration (43,300 mM) [5]<br>W_{wm} = pure WM concentration (35,880 mM) [5]
**4. Data quality**	
a. Reported variables (SNR, linewidth with reference peaks)	SNR: Ratio of N-acetyl aspartate peak at 2ppm to measured noise variance<br>Water linewidth<br>(See Supplementary Table 3)
b. Data exclusion criteria	SNR < 5<br>Water Linewidth > 0.1 ppm (12.8 Hz)<br>Issues with voxel placement<br>Z score of main metabolite concentration > 3
c. Quality measures of postprocessing model fitting (e.g., CRLB, goodness of fit, SD of residual)	Cramér Rao Lower Bounds (CRLB)<br>(See Supplementary Table 3)
d. Sample spectrum	See Supplementary Figure 1

Figure 2: Exemplary fitting of J-modulated Sample spectra.

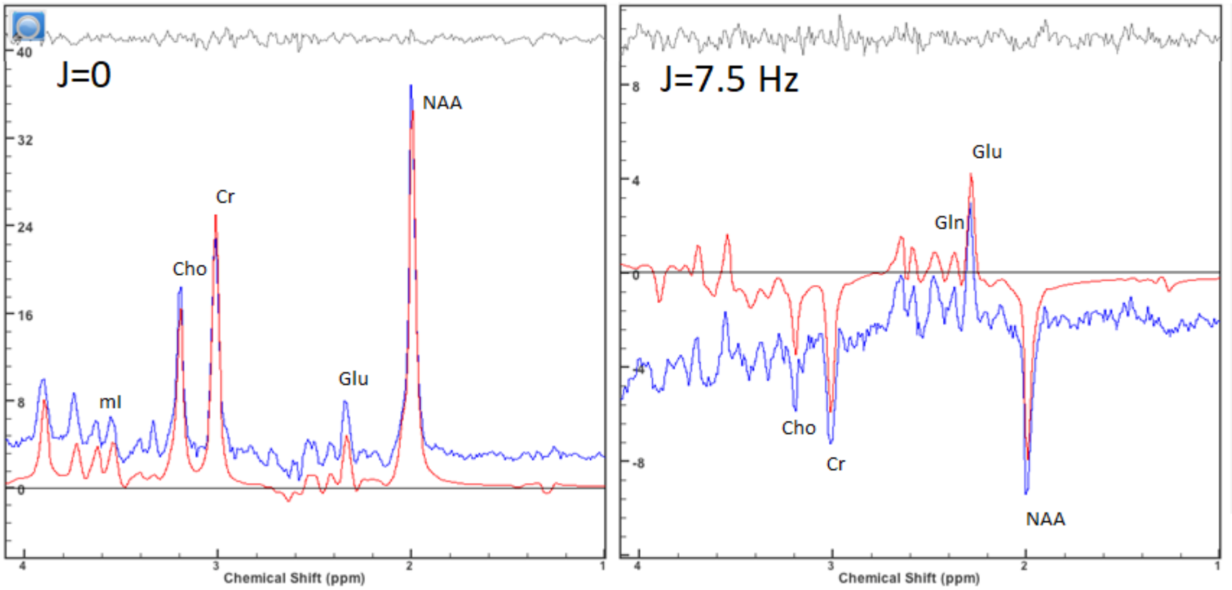

Showcase of the fitting results of J-modulated spectra, left: J = 0 and right: J = 7.5 Hz. The fitted spectra (red) are vertically shifted from the input spectra (blue) for better visualization, and the fit residuals are shown in grey. Glutamate detection is enhanced by combining the two J-modulated spectra.

Abbreviations: Cho, Choline; Cr, Creatine; Gln: Glutamine; Glu: Glutamate; Hz: Hertz; mI: Myo-inositol; NAA: N-acetyl aspartate.

Table 2. Model determination: Checking the influence of age on glutamate concentration.

	Persistent ADHD	Remitting ADHD	Never Affected
Glutamate ~ Age at scan	**255.8**	**85.14**	**294.78**
Glutamate ~ (Age at scan)^2	257.53	87.1	296.27
Glutamate ~ (Age at scan)^3	259.1	88.8	296.05
Glutamate ~ Age at scan + sex + CRLB	**255.21**	**88.95**	**298.41**
Glutamate ~ (Age at scan)^2 + sex + CRLB	256.88	90.9	299.64
Glutamate ~ (Age at scan)^3 + sex + CRLB	258.33	92.57	299.39

Akaike information criterion (AIC) of the different linear mixed models (including age at scan only and full model) by outcome group. Best AIC in bold.

Abbreviation: CRLB, Cramér Rao Lower Bounds.

Supplementary Results

Figure 3. Averaged MRS Spectra by Outcome Group and Individual participant's spectra.

A) Average Spectra.

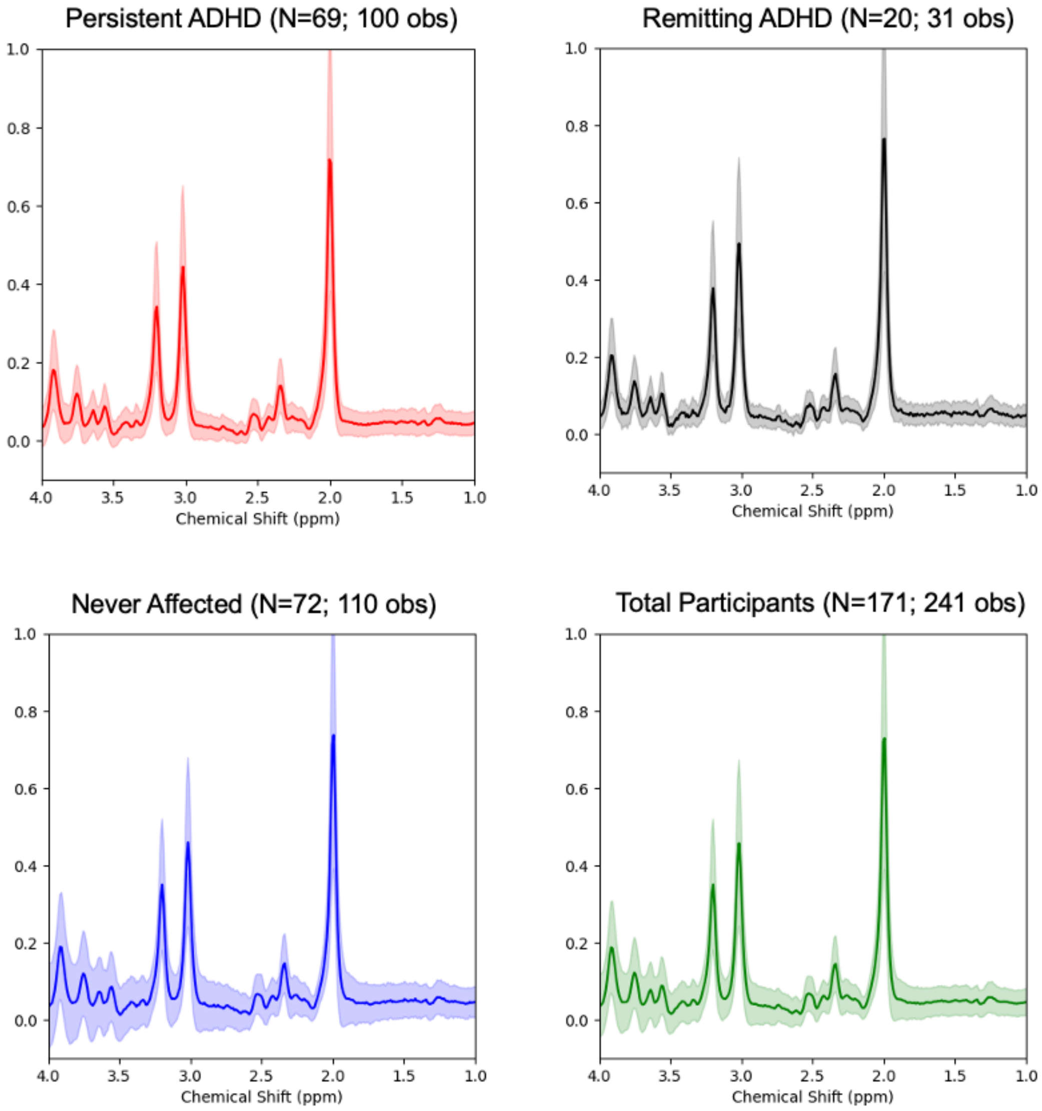

Fitted averaged spectra by outcome groups and average spectrum across all participants, shaded regions represent the standard deviation. Prior to averaging, each spectrum was scaled to its water reference amplitude, phase-corrected, and aligned to correct for frequency offsets. Variances in spectral width and baseline account for a substantial portion of the overall spectral deviations.

B) Individual participant's spectrum by outcome group.

- Persistent ADHD (N=69, 100 observations)

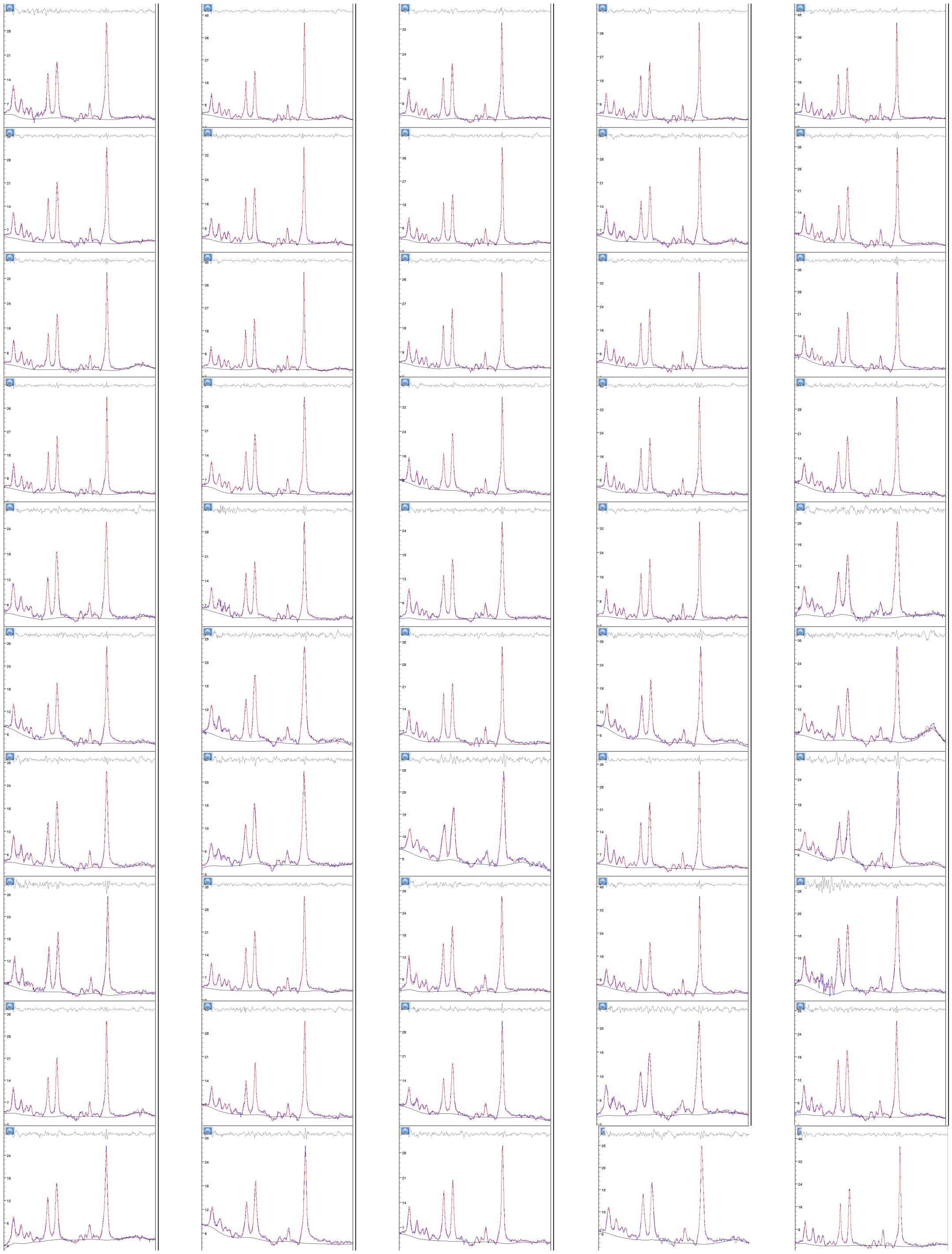

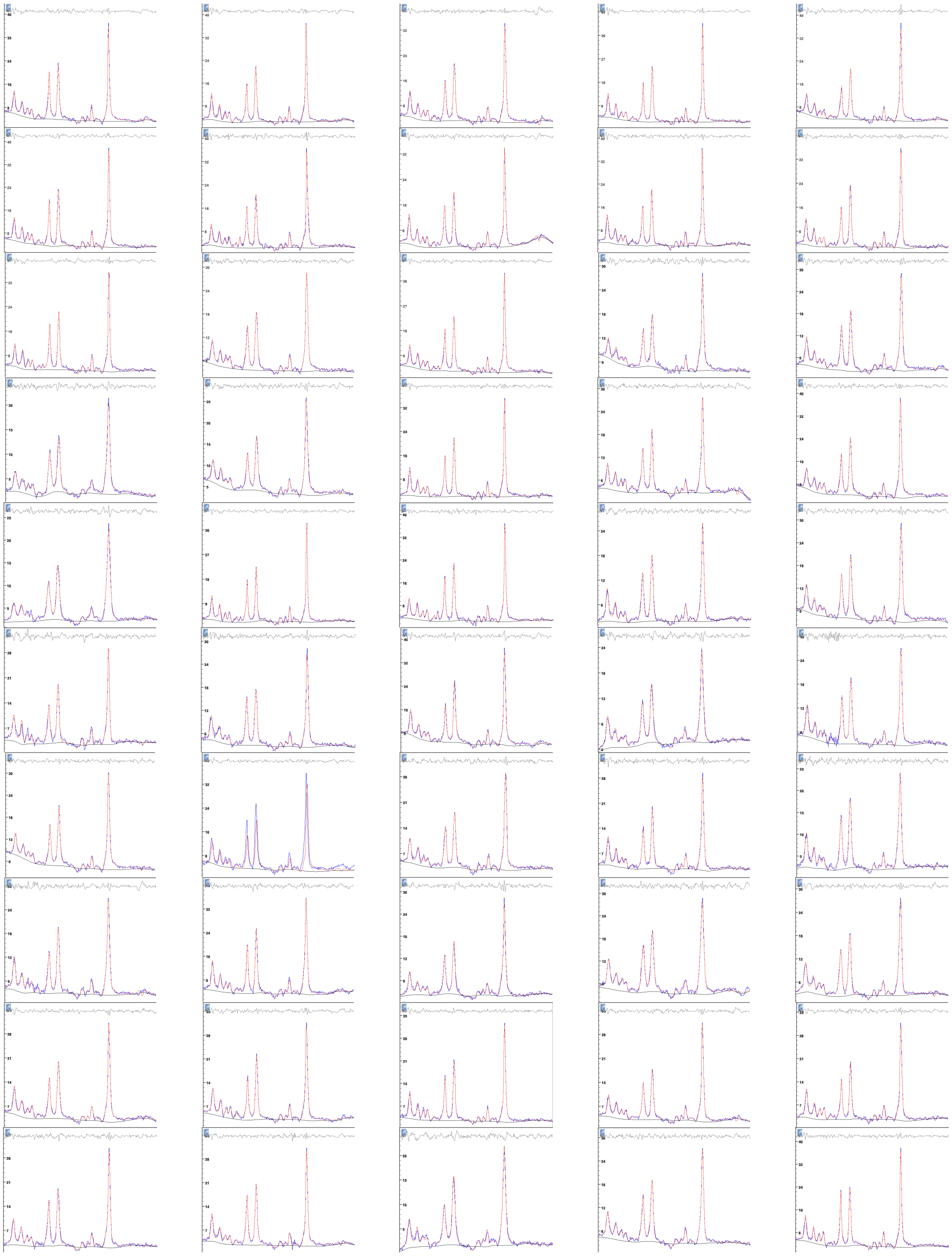

- Remitting ADHD (N=20, 31 observations)

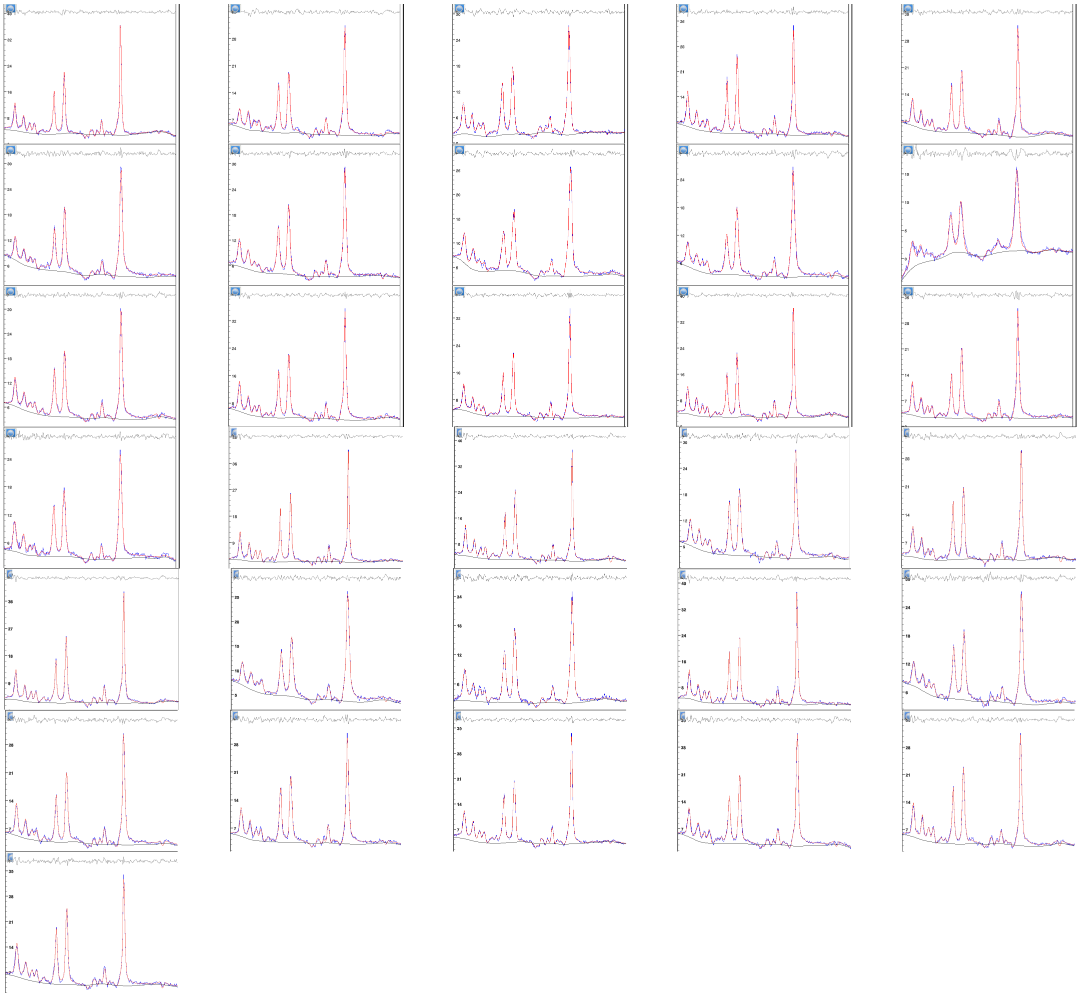

- Never Affected (N=72, 110 observations)

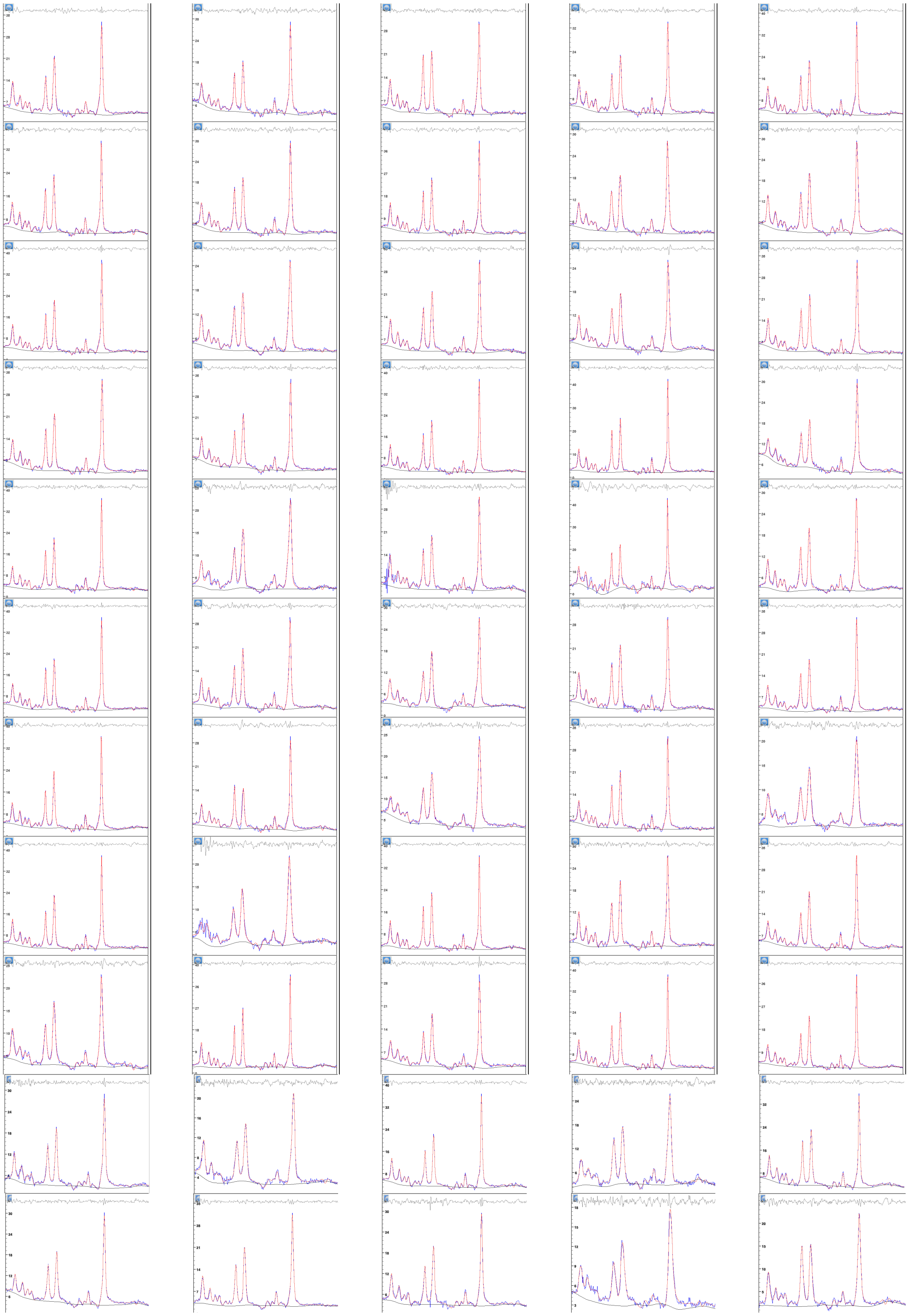

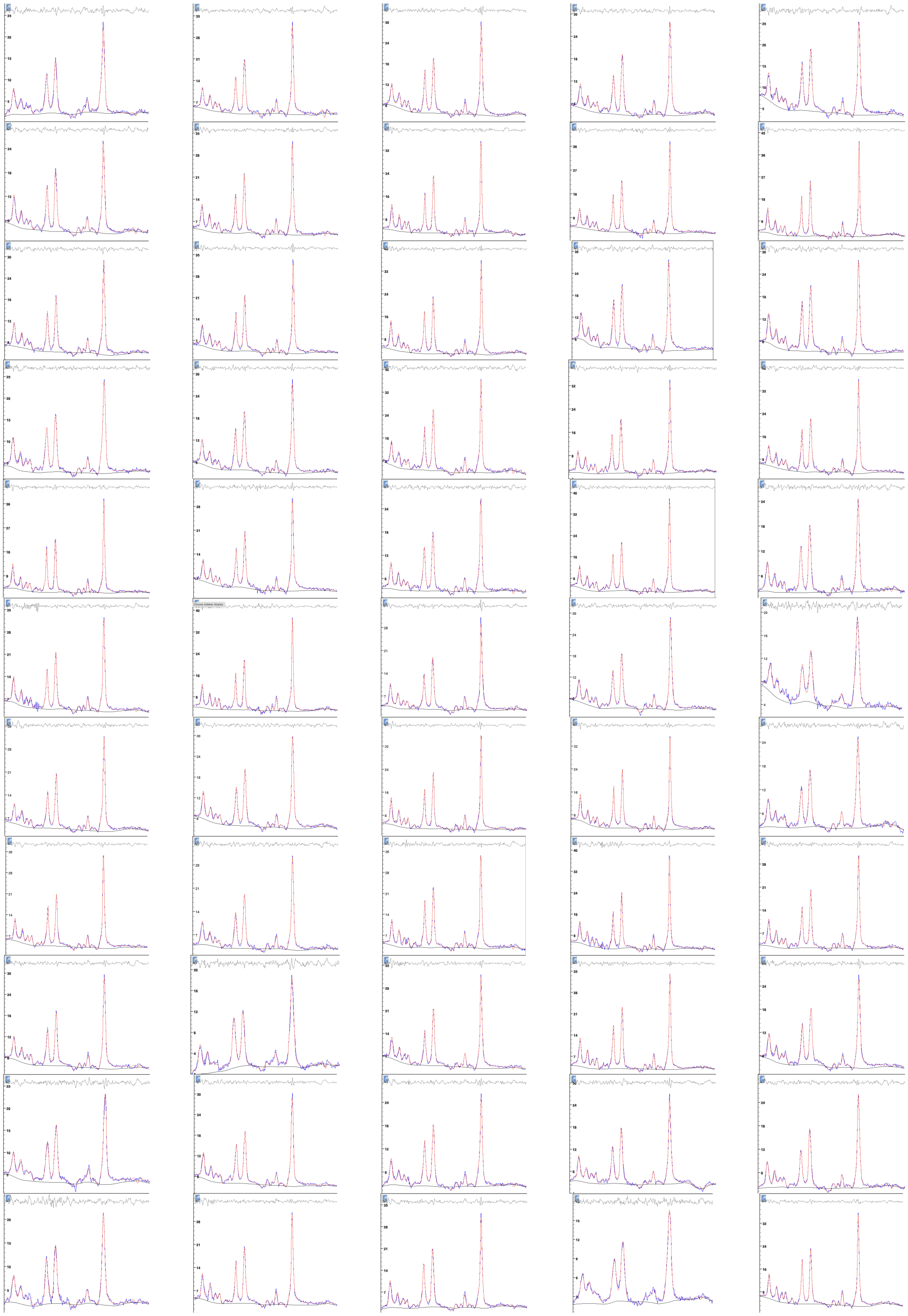

Figure 4. Flow chart of data included.

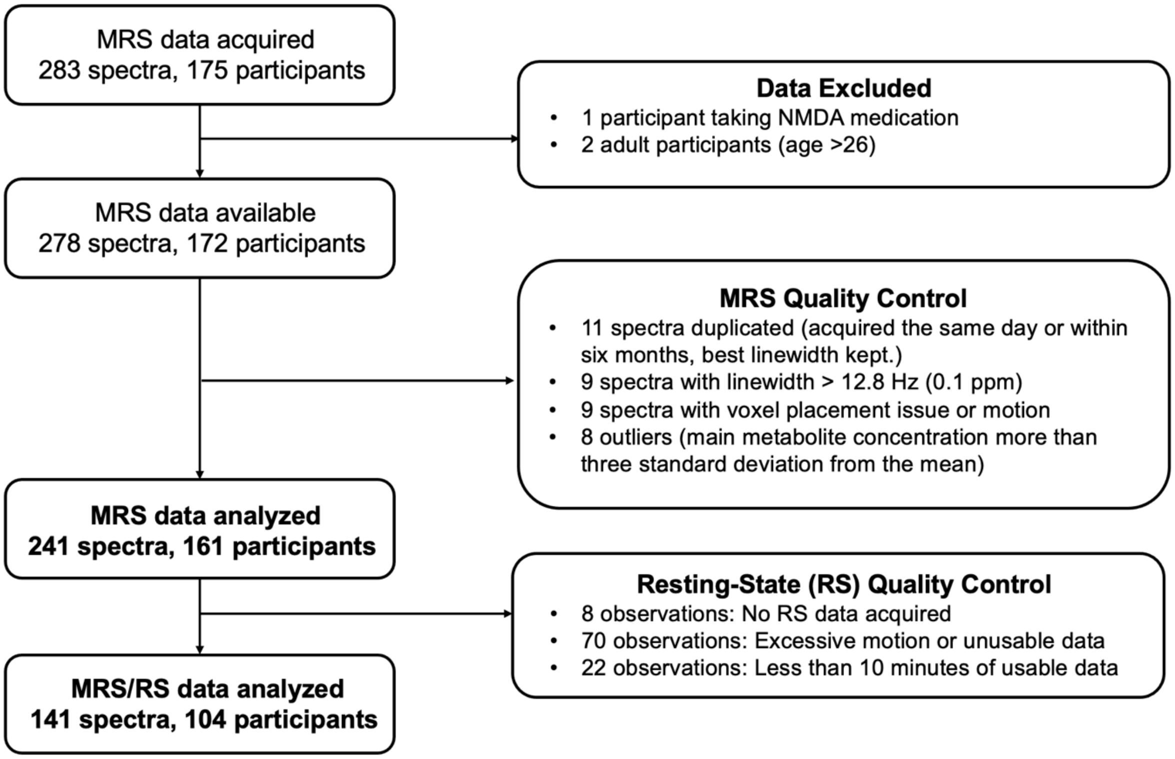

Abbreviations: MRS, magnetic resonance spectroscopy; NMDA, N-methyl-D-aspartate -NMDA medication is a drug that binds to the NMDA glutamate receptor; RS, Resting-state fMRI data.

Table 3. Data Quality Control.

	Persistent ADHD N=69 100 obs	Remitting ADHD N=20 31 obs	Never Affected N=72 110 obs	Statistics
**Magnetic resonance spectroscopy: Voxel Composition**				
**Percent of grey matter**	63.22 [50.47;71.72]	62.99 [57.4;70.15]	64.38 [52.51;70.18]	K-W$\chi^2_{(2)}$= 1.77 p-value = 0.41
**Percent of white matter**	18.19 [10.23;27.44]	18.16 [13.63;25.51]	18.08 [10.93;34.15]	K-W$\chi^2_{(2)}$= 0.62 p-value = 0.73
**Percent of cerebrospinal fluid**	17.7 [10.9;30.82]	18.17 [13.1;26.94]	17.43 [10.23;36.56]	K-W$\chi^2_{(2)}$ = 1.15 p-value = 0.56
**Magnetic resonance spectroscopy: Quality Metrics**				
**Linewidth** (Hz)	8.18 [4.91;11.4]	7.94 [5.18;11.45]	8.18 [4.93;12.45]	K-W$\chi^2_{(2)}$ = 0.31 p-value = 0.85
**Signal-to-noise ratio**	38.04 [16.05;78.55]	38.07 [16.18;69.23]	42.13 [18.61;97.85]	K-W$\chi^2_{(2)}$ = 1.84 p-value = 0.39
**Glutamate CRLB** (%)	6.86 [3.87;13.85]	6.76 [3.6;13.83]	6.61 [3.45;23.67]	K-W$\chi^2_{(2)}$ = 0.09 p-value = 0.96
**Total N-acetyl aspartate CRLB** (%)	1.88 [1.21;3.5]	1.81 [1.06;3.34]	1.79 [1.14;4.23]	K-W$\chi^2_{(2)}$ = 0.36 p-value = 0.84
**Resting-State fMRI: Quality Metrics**				
**Number of participants and observations**	N=39 49 obs	N=16 19 obs	N=49 73 obs	-
**Mean framewise displacement*** (mm)	0.12 [0.06;0.25]	0.1 [0.06;0.24]	0.13 [0.05;0.27]	K-W$\chi^2_{(2)}$ = 0.31 p-value = 0.86

Data are presented as median [range].
*The mean framewise displacement refers to the average relative root mean square of head motion as defined by Jenkinson *et al*. [6].
Abbreviations: CRLB, Cramér Rao Lower Bounds; obs, number of observations.

Table 4. Glutamate at baseline: Cross-sectional Analysis.

	Statistics	P-value
**Sex**	$F_{(1)}$=0.87	0.35
**Glutamate CRLB**	$F_{(1)}$=0.02	0.89
**Age at Scan**	$F_{(1)}$=0.01	0.35
**Outcome group**	$F_{(2)}$=0.86	0.42

Linear regression F-test.
Abbreviation: CRLB, Cramér Rao Lower Bounds.

Table 5. Developmental Glutamate, Glutamine and “Glx” Analyses.

	**Glutamate**		**Glutamine**		**Glx**		**Glutamate/Total Creatine**	
Fixed Effect	Statistics	P-value	Statistics	P-value	Statistics	P-value	Statistics	P-value
Outcome group	$F_{(2,157)}$=2.78	0.06	$F_{(2,157)}$=0.1	0.91	$F_{(2,157)}$=1.47	0.23	$F_{(2,157)}$ = 0.52	0.6
Age at scan	$F_{(1,76)}$=5.07	0.03	$F_{(1,76)}$=5.06	0.03	$F_{(1,76)}$=3.42	0.07	$F_{(1,76)}$ =22.92	$8.13x10^{-6}$
Sex	$F_{(1,157)}$=1.96	0.16	$F_{(1,157)}$=1.82	0.18	$F_{(1,157)}$=0.56	0.45	$F_{(1,157)}$=0.43	0.51
CRLB	$F_{(1,76)}$=0.21	0.64	$F_{(1,76)}$=17.49	$5.6x10^{-4}$	$F_{(1,76)}$=0.86	0.36	$F_{(1,76)}$=10.33	$2x10^{-3}$
**Outcome group*age**	**$F_{(2,76)}$=3.89**	**0.02**	$F_{(2,76)}$=1.38	0.26	**$F_{(2,76)}$=5.46**	**$6x10^{-3}$**	$F_{(2,76)}$=0.88	0.42

Linear Mixed Model F-test.
Abbreviations: CRLB, Cramér Rao Lower Bounds; Glx, glutamate and glutamine.

		**Persistent ADHD**	**Remitting ADHD**	**Never Affected**
**Glutamate**	Estimate (SE) [95% bootstrapped CI] *Std. Estimate [95% CI]*	0.096 (0.04) [0.01;0.18] *0.32 [0.04, 0.60]*	-0.072 (0.07) [-0.21;0.08] *-0.24 [-0.76, 0.27]*	-0.10 (0.04) [-0.18, -0.01] *-0.32 [-0.60, -0.04]*
	Statistics – *Ref: Never affected*	**$t_{(76)}$=2.28; p=0.026**	$t_{(76)}$=-0.94 ;p=0.35	
	Statistics – *Ref: Persistent ADHD*		**$t_{(76)}$= -2.18; p=0.032**	
**Glx**	Estimate (SE) [95% bootstrapped CI] *Std. Estimate [95% CI]*	0.14 (0.05) [0.04;0.25] *0.38 [0.11, 0.65]*	-0.09 (0.09) [-0.24;0.07] *-0.25 [-0.75, 0.25]*	-0.14 (0.05) [-0.25, -0.04] *-0.38 [-0.65, -0.11]*
	Statistics – *Ref: Never affected*	**$t_{(76)}$ = 2.76; p = 0.007**	$t_{(71)}$ = -0.99, p = 0.33	
	Statistics – *Ref: Persistent ADHD*		**$t_{(71)}$=-2.26; p=0.027**	

Glutamate and Glx: β estimate (mM/year) of the interaction between outcome group and age from the linear mixed model.

Table 6. Developmental Glutamate: Sensitivity analyses and robustness checks.

A) Intellectual Quotient (IQ), psychostimulant medication and comorbidities.

Model	Fixed effect: Outcome group * age		**Persistent ADHD**	**Remitting ADHD**	**Never Affected**
**IQ Covariate**	**$F_{(2,76)}$=3.87 p=0.025**	Estimate (SE)	0.095 (0.04)	-0.076 (0.07)	-0.10 (0.04)
		[95% bt CI]	[0.01;0.17]	[-0.21;0.08]	[-0.18, -0.01]
		Std. Estimate [95% CI]	*0.32 [0.04, 0.60]*	*-0.24 [-0.76, 0.27]*	*-0.32 [-0.60, -0.04]*
		Statistics – *Ref: Never affected*	**$t_{(76)}$=2.27 p=0.026**	$t_{(76)}$=-0.94 p=0.34	
		Statistics – *Ref: Persistent ADHD*		**$t_{(76)}$= -2.18 p=0.032**	
**Psychostimulant Medication at the time of neuroimaging Covariate**	**$F_{(2,75)}$=3.86 p=0.025**	Estimate (SE)	0.096 (0.04)	-0.072 (0.08)	-0.10 (0.04)
		[95% bt CI]	[0.02;0.17]	[-0.21;0.07]	[-0.18, -0.01]
		Std. Estimate [95% CI]	*0.32 [0.04, 0.61]*	*-0.24 [-0.76, 0.27]*	*-0.32 [-0.61, -0.04]*
		Statistics – *Ref: Never affected*	**$t_{(75)}$=2.26 p=0.027**	$t_{(75)}$ = -0.94 p=0.350	
		Statistics – *Ref: Persistent ADHD*		**$t_{(75)}$=2.26; p=0.027**	
**Ever on Psychostimulant Medication Covariate**	**$F_{(2,76)}$=3.93 p=0.024**	Estimate (SE)	0.098 (0.04)	-0.07 (0.08)	-0.098 (0.04)
		[95% bt CI]	[0.02, 0.17]	[-0.21;0.09]	[-0.17, -0.02]
		Std. Estimate [95% CI]	*0.33 [0.05, 0.61]*	*-0.23 [-0.75, 0.29]*	*-0.33 [-0.61, -0.05]*
		Statistics – *Ref: Never affected*	**$t_{(76)}$=2.31 p=0.023**	$t_{(76)}$ = -0.88 p=0.383	
		Statistics – *Ref: Persistent ADHD*		**$t_{(76)}$=-2.15; p=0.035**	
**Excluding Comorbidities**	**$F_{(2,71)}$=4.07 p=0.021**	Estimate (SE)	0.01 (0.04)	-0.07 (0.08)	-0.11 (0.04)
		[95% bt CI]	[0.02;0.19]	[-0.22;0.08]	[-0.19, -0.02]
		Std. Estimate [95% CI]	*0.35 [0.05, 0.65]*	*-0.24 [-0.75, 0.27]*	*-0.35 [-0.65, -0.05]*
		Statistics – *Ref: Never affected*	**$t_{(71)}$=2.36; p=0.021**	$t_{(71)}$ = -0.94, p = 0.35	
		Statistics – *Ref: Persistent ADHD*		**$t_{(71)}$=-2.26; p=0.027**	

Abbreviations: bt: bootstrapped; CI: Confidence interval; SE: Standard error; Std: Standardized.

B) Analysis of sex specific effect.

Linear Mixed Model: $Glutamate_{ij}$ ~ $outcome\_group_i * age_{ij} * sex_i + glutamate\_CRLB_{ij}$,
Random intercept: 1| Participant

The model did not converge with using the three outcome groups (Persistent ADHD, Remitting ADHD, and Never Affected). Thus, the results provided below include only the two main outcome groups: Persistent ADHD *vs.* Never Affected.

	Statistics	P-value
**Outcome group**	$F_{(1,137)}$=1.88	0.17
**Age at Scan**	$F_{(1,64)}$=1.79	0.19
**Sex**	$F_{(1,137)}$=0.06	0.8
**Glutamate CRLB**	$F_{(1,64)}$=0.53	0.46
**Outcome group*Age at scan**	$F_{(1,64)}$=2.96	0.09
**Outcome group*Sex**	$F_{(1,137)}$=0.41	0.52
**Age at scan*Sex**	$F_{(1,64)}$=0.32	0.57
**Outcome group*Age at scan*Sex**	$F_{(1,64)}$=0.76	0.39

Abbreviation: CRLB, Cramér Rao Lower Bounds.

C) Glutamate analysis without the remitting group.

Linear Mixed Model with two outcome groups only: Persistent ADHD vs. Never Affected (Reference group).
$Glutamate_{ij}$ ~ $outcome\_group_i * age_{ij} * sex_i + glutamate\_CRLB_{ij}$,
Random intercept: 1| Participant

	**Glutamate**		
	Std. Estimate [95%CI]	Statistics	P-value
**Intercept**	10.73 [9.85, 11.61]	$t_{(138)}$ = 24.14	**< .001**
**Outcome group**	0.18 [-0.12, 0.49]	$t_{(138)}$ = -1.94	0.054
**Age at scan**	-0.09 [-0.30, 0.12]	$t_{(66)}$ = -0.84	0.402
**Sex**	-0.24 [-0.58, 0.10]	$t_{(138)}$ = -1.41	0.161
**CRLB**	-0.05 [-0.19, 0.10]	$t_{(66)}$ = -0.63	0.53
**Outcome group*age**	**0.33 [0.04, 0.63]**	**$t_{(66)}$ = 2.28**	**0.026**

Abbreviations: CRLB, Cramér Rao Lower Bounds; CI: Confidence interval; Std: Standardized.

Table 7. Relationship between outcome group and MRS metabolites.

	Total N-acetyl aspartate		Total Choline		Total Creatine		GABA	
	Statistics	P-value	Statistics	P-value	Statistics	P-value	Statistics	P-value
Outcome group	$F_{(2,157)}=3.46$	0.03	$F_{(2,157)}=0.09$	0.91	$F_{(2,157)}=0.19$	0.83	$F_{(2,156)}=1.99$	0.14
Age at scan	$F_{(1,76)}=18.65$	0.003	$F_{(1,76)}=9.43$	0.003	$F_{(1,76)}=28.03$	$2x10^{-4}$	$F_{(1,76)}=8.1$	$5x10^{-3}$
Sex	$F_{(1,157)}=0.75$	0.39	$F_{(1,157)}=7.75$	0.01	$F_{(1,157)}=0.61$	0.44	$F_{(1,156)}=2x10^{-3}$	0.96
CRLB^	$F_{(1,76)}=2.86$	0.09	$F_{(1,76)}=29.82$	$6x10^{-7}$	$F_{(1,76)}=30.59$	$5x10^{-7}$	$F_{(1,76)}=1.44$	0.23
Outcome group*age	$F_{(2,76)}=3.91$	0.02	$F_{(2,76)}=1.61$	0.21	$F_{(2,76)}=2.23$	0.11	$F_{(2,76)}=2.11$	0.13

Linear Mixed Model F-test.
^ GABA analysis: the quantification technique used is not specific to GABA and no CRLB were provided; instead, water linewidth was used in the model to control for data quality.
Abbreviations: CRLB, Cramér Rao Lower Bounds; GABA: Gamma-aminobutyric acid.

		**Persistent ADHD**	**Remitting ADHD**	**Never Affected**
**Total N-acetyl aspartate**	Estimate (SE) [95% bootstrapped CI] *Std. Estimate [95% CI]*	0.07 (0.03) [0.006; 0.13] *0.32 [0.04, 0.60]*	-0.04 (0.05) [-0.15; 0.07] *-0.24 [-0.76, 0.27]*	0.02 (0.02) [-0.2; 0.07] *-0.32 [-0.60, -0.04]*
	Statistics – *Ref: Never affected*	**$t_{(76)}=2.35$ p=0.02**	$t_{(76)}=-0.8$ p=0.42	
	Statistics – *Ref: Persistent ADHD*		**$t_{(76)}=2.1$ p=0.04**	

Total N-acetyl aspartate: β estimate (mM/year) of the interaction between outcome group and age from the linear mixed model.

Table 8. Resting-State Networks, Glutamate, and Outcome group.

A) Primary analysis

	**Within DMN**		**DMN-dorsal attention network**		**DMN-central executive network**		**DMN-salience network**		**DMN-subcortical**	
	Std. Estimate [95%CI]	Statistics, p-value	Std. Estimate [95%CI]	Statistics, p-value	Std. Estimate [95%CI]	Statistics, p-value	Std. Estimate [95%CI]	Statistics, p-value	Std. Estimate [95%CI]	Statistics, p-value
Intercept	0.42 [0.25,0.62]	**$t_{(85)}$=5.03 p=3x10^{-6}**	-0.2 [-0.36;-0.01]	**$t_{(85)}$=-2.04 p=0.04**	-0.06 [-0.2;0.08]	$t_{(85)}$=-0.72 p=0.48	-0.14 [-0.31;0.02]	$t_{(85)}$=-1.4 p=0.17	0.14 [0.01;0.26]	$t_{(85)}$=1.99 p=0.05
Glutamate	-0.34 [-0.56,-0.12]	**$t_{(29)}$=3.17 p=4x10^{-3}**	0.14 [-0.07, 0.35]	$t_{(29)}$=1.37 p=0.18	0.13 [-0.07, 0.33]	$t_{(29)}$=1.29 p=0.21	0.1 [-0.10, 0.30]	$t_{(29)}$=1.05 p=0.3	-0.19 [-0.41, 0.03]	$t_{(29)}$=-1.74 p=0.09
Outcome group	0.15 [-0.25, 0.54]	**$t_{(85)}$=-2.45 p=0.02**	0.02 [-0.36, 0.40]	$t_{(85)}$=-0.12 p=0.91	0.27 [-0.07, 0.62]	**$t_{(85)}$=-2.13 p=0.04**	0.01 [-0.38, 0.40]	$t_{(85)}$=-0.43 p=0.67	-0.25 [-0.62, 0.12]	**$t_{(85)}$=-2.88 p=5x10^{-3}**
Sex	-0.2 [-0.63,0.22]	$t_{(85)}$=-0.94 p=0.35	0.29 [-0.12, 0.71]	$t_{(85)}$=1.4 p=0.16	-0.22 [-0.59, 0.16]	$t_{(85)}$=-1.13 p=0.26	0.11 [-0.31, 0.53]	$t_{(85)}$=0.51 p=0.61	0.36 [-0.05, 0.76]	$t_{(85)}$=1.76 p=0.08
Motion	-0.04 [-0.23, 0.16]	$t_{(29)}$=0.08 p=0.93	0.36 [0.18, 0.55]	$t_{(29)}$=0.47 p=0.64	0.33 [0.15, 0.51]	$t_{(29)}$=0.25 p=0.81	0.4 [0.23, 0.58]	$t_{(29)}$=1.17 p=0.25	0.23 [0.03, 0.42]	$t_{(29)}$=1.41 p=0.17
Motion^2	-0.01 [-0.16, 0.13]	$t_{(29)}$=-0.19 p=0.85	0.03 [-0.11, 0.17]	$t_{(29)}$=0.44 p=0.66	0.04 [-0.09, 0.18]	$t_{(29)}$=0.62 p=0.53	-0.01 [-0.14, 0.12]	$t_{(29)}$=-0.16 p=0.87	-0.07 [-0.22, 0.07]	$t_{(29)}$=-1.01 p=0.32
Age at scan	0.11 [-0.08, 0.30]	$t_{(29)}$=1.15 p=0.26	-0.04 [-0.23, 0.14]	$t_{(29)}$=-0.48 p=0.63	0.02 [-0.15, 0.19]	$t_{(29)}$=0.23 p=0.82	-0.13 [-0.31, 0.05]	$t_{(29)}$=-1.51 p=0.14	-0.14 [-0.33, 0.04]	$t_{(29)}$=-1.57 p=0.13
Glutamate* Outcome group	0.5 [0.10, 0.91]	**$t_{(29)}$=2.53 p=0.017**	0.02 [-0.36, 0.41]	$t_{(29)}$=0.13 p=0.9	0.41 [0.04, 0.78]	**$t_{(29)}$=2.28 p=0.03**	0.08 [-0.30, 0.46]	$t_{(29)}$=0.44 p=0.66	0.54 [0.14, 0.94]	**$t_{(29)}$=2.78 p=0.009***
Glutamate* Outcome group[IQ]	0.51 [0.10, 0.92]	**$t_{(29)}$=2.54 p=0.017**	0.03 [-0.36, 0.42]	$t_{(29)}$=0.14 p=0.889	0.43 [0.06, 0.80]	**$t_{(29)}$=2.39 p = 0.024**	0.06 [-0.32, 0.44]	$t_{(29)}$= 0.33 p = 0.745	0.54 [0.14, 0.94]	**$t_{(29)}$= 2.75, p = 0.01**
Glutamate* Outcome group[Stim]	0.51 [0.10, 0.91]	**$t_{(28)}$=2.54 p 0.017**	0.03 [-0.37, 0.42]	$t_{(28)}$=0.13p = 0.896	0.41 [0.04, 0.79]	**$t_{(28)}$=2.28 p = 0.03**	0.08 [-0.30, 0.46]	$t_{(28)}$=0.43p = 0.670	0.55 [0.15, 0.94]	**$t_{(28)}$=2.81 p = 0.009***

Glutamate* Outcome group[StimEver]	0.51 [0.10, 0.92]	**$t_{(29)}$=2.54 p 0.017**	-1.17x10^{-3} [-0.39, 0.39]	$t_{(29)}$=-6.1 x10^{-3} p = 0.995	0.40 [0.03, 0.77]	**$t_{(29)}$=2.19 p = 0.037**	0.06 [-0.32, 0.43]	$t_{(29)}$=0.33 p = 0.74	0.54 [0.14, 0.94]	**$t_{(29)}$=2.74 p = 0.01**
Glutamate* Outcome group[Co-Dx]	0.57 [0.12, 1.01]	**$t_{(27)}$=2.63 p=0.014**	-0.04 [-0.47, 0.38]	$t_{(27)}$= -0.20 p = 0.842	0.45 [0.05, 0.85]	**$t_{(27)}$= 2.31 p=0.028**	0.05 [-0.37, 0.47]	$t_{(27)}$= 0.23, p = 0.817	0.51 [0.08, 0.93]	**$t_{(27)}$=2.43 p = 0.022**

Linear Mixed Model: $Network\_metric_{ij} \sim Glutamate_{ij} * outcome\ group_i + age_{jj} + sex_i + motion_{ij} + (motion_{ij})^2$
Random intercept: 1| Participant

Linear Mixed Model with two outcome groups only: Persistent ADHD vs. Never Affected (Reference group).
[IQ]: Model with full scale intellectual quotient (IQ) added as a covariate
[Stim]: Model with psychostimulant medication status at the time of MRI added as a covariate (participants were off psychostimulant the day of the scan, however, we kept tracked off ongoing psychostimulant therapy).
[StimEver]: Model with covariate “ever received psychostimulant treatment” added.
[Co-Dx]: Same model excluding 13 participants with comorbidities.

B) Post-Hoc Analyses: Investigating the subcortical divisions.

	**DMN-Caudate**	**DMN-Dorsal Putamen**	**DMN-Ventral Putamen**	**DMN-Thalamus**	**DMN-Ventral Striatum**	**DMN-Dorsal Amygdala**	**DMN-Ventral Amygdala**
	Std. Estimate [95%CI]<br>Statistics<br>p-value	Std. Estimate [95%CI]<br>Statistics<br>p-value	Std. Estimate [95%CI]<br>Statistics<br>p-value	Std. Estimate [95%CI]<br>Statistics<br>p-value	Std. Estimate [95%CI]<br>Statistics<br>p-value	Std. Estimate [95%CI]<br>Statistics<br>p-value	Std. Estimate [95%CI]<br>Statistics<br>p-value
Intercept	0.25 [0.05, 0.46]<br>$t_{(85)} = 2.43$<br>p = 0.017	-7.22x10^{-4}[-0.22,0.22]<br>$t_{(85)} = -6.50x10^{-3}$<br>p = 0.995	-0.02 [-0.26, 0.22]<br>$t_{(85)} = -0.15$<br>p = 0.878	0.15 [-0.10, 0.39]<br>$t_{(85)} = 1.17$<br>p = 0.244	0.05 [-0.18, 0.28]<br>$t_{(85)} = 0.43$<br>p = 0.669	0.28 [0.09, 0.47]<br>$t_{(85)} = 2.92$<br>p = 0.005	0.31 [0.10, 0.52]<br>$t_{(85)} = 2.89$<br>p = 0.005
Glutamate	-0.25 [-0.46, -0.03]<br>$t_{(29)} = -2.34$<br>p = 0.026	-0.07 [-0.28, 0.15]<br>$t_{(29)} = -0.62$<br>p = 0.538	0.01 [-0.21, 0.24]<br>$t_{(29)} = 0.12$<br>p = 0.908	-0.08 [-0.30, 0.14]<br>$t_{(29)} = -0.71$<br>p = 0.483	0.03 [-0.20, 0.26]<br>$t_{(29)} = 0.23$<br>p = 0.819	-0.32 [-0.54, -0.10]<br>$t_{(29)} = -2.97$, p = 0.006	-0.26 [-0.48, 0.03]<br>$t_{(29)} = -2.30$<br>p = 0.029
Outcome group	-0.33 [-0.69, 0.02]<br>$t_{(85)} = -3.85$<br>p < .001	-0.19 [-0.57, 0.18]<br>$t_{(85)} = -2.00$<br>p = 0.049	-0.11 [-0.48, 0.26]<br>$t_{(85)} = -1.77$<br>p = 0.080	-0.17 [-0.55, 0.21]<br>$t_{(85)} = -1.15$<br>p = 0.254	0.10 [-0.31, 0.51]<br>$t_{(85)} = -0.48$<br>p = 0.631	-0.37 [-0.76, 0.02]<br>$t_{(85)} = -2.46$, p = 0.016	-0.14 [-0.54, 0.27]<br>$t_{(85)} = -2.00$<br>p = 0.049
Sex	0.28 [-0.11, 0.67]<br>$t_{(85)} = 1.44$<br>p = 0.154	0.22 [-0.19, 0.63]<br>$t_{(85)} = 1.07$<br>p = 0.288	0.25 [-0.15, 0.65]<br>$t_{(85)} = 1.26$<br>p = 0.211	0.50 [0.09, 0.91]<br>$t_{(85)} = 2.42$<br>p = 0.018	0.17 [-0.28, 0.61]<br>$t_{(85)} = 0.74$<br>p = 0.463	0.12 [-0.30, 0.54]<br>$t_{(85)} = 0.57$<br>p = 0.572	0.04 [-0.40, 0.48]<br>$t_{(85)} = 0.17$<br>p = 0.866
Motion	0.22 [0.03, 0.41]<br>$t_{(29)} = 1.10$<br>p = 0.279	0.34 [0.14, 0.53]<br>$t_{(29)} = 2.10$<br>p = 0.044	0.27 [0.07, 0.47]<br>$t_{(29)} = 1.19$<br>p = 0.244	0.09 [-0.11, 0.28]<br>$t_{(29)} = 1.09$<br>p = 0.286	0.03 [-0.18, 0.23]<br>$t_{(29)} = 0.47$<br>p = 0.645	0.09 [-0.11, 0.28]<br>$t_{(29)} = 0.70$<br>p = 0.490	-0.03 [-0.23, 0.17]<br>$t_{(29)} = -0.28$<br>p = 0.785
Motion^2	-0.05 [-0.19, 0.10]<br>$t_{(29)} = -0.66$<br>p = 0.516	-0.10 [-0.25, 0.04]<br>$t_{(29)} = -1.49$<br>p = 0.148	-0.05 [-0.19, 0.10]<br>$t_{(29)} = -0.65$<br>p = 0.523	-0.07 [-0.22, 0.07]<br>$t_{(29)} = -0.99$<br>p = 0.330	-0.03 [-0.19, 0.12]<br>$t_{(29)} = -0.46$<br>p = 0.650	-0.04 [-0.19, 0.11]<br>$t_{(29)} = -0.56$<br>p = 0.578	0.01 [-0.19, 0.22]<br>$t_{(29)} = -0.24$<br>p = 0.816
Age at scan	-0.08 [-0.26, 0.10]<br>$t_{(29)} = -0.92$<br>p = 0.367	-0.13 [-0.32, 0.05]<br>$t_{(29)} = -1.46$<br>p = 0.155	-0.13 [-0.32, 0.05]<br>$t_{(29)} = -1.46$<br>p = 0.154	-0.26 [-0.45, -0.07]<br>$t_{(29)} = -2.76$<br>p = 0.010	-4.22x10^{-4} [-0.20, 0.20]<br>$t_{(29)} = -4.27x10^{-3}$<br>p = 0.997	-6.55x10^{-3} [-0.20, 0.19]<br>$t_{(29)} = -0.07$<br>p = 0.945	0.02 [-0.13, 0.17]<br>$t_{(29)} = 0.29$<br>p = 0.771
Glutamate* Outcome group	**0.70 [0.31, 1.09]**<br>**$t_{(29)} = 3.71$**<br>**p =0.0009**	0.37 [-0.02, 0.77]<br>$t_{(29)} = 1.92$<br>p = 0.065;	0.34 [-0.06, 0.74]<br>$t_{(29)} = 1.73$<br>p = 0.095	0.21 [-0.19, 0.61]<br>$t_{(29)} = 1.07$<br>p = 0.292	0.11 [-0.32, 0.54]<br>$t_{(29)} = 0.53$<br>p = 0.600	**0.46 [0.05, 0.86]**<br>**$t_{(29)} = 2.30$**<br>**p = 0.029**	0.40 [-0.02, 0.82]<br>$t_{(29)} = 1.94$<br>p = 0.062
Glutamate* Outcome group[IQ]	**0.67 [0.25, 1.08]**<br>**$t_{(19)} = 3.35$**<br>**p = 0.003**	0.37 [-0.06, 0.80]<br>$t_{(19)} = 1.79$<br>p = 0.089	0.34 [-0.09, 0.77]<br>$t_{(19)} = 1.64$<br>p = 0.117	0.22 [-0.21, 0.65]<br>$t_{(19)} = 1.08$<br>p = 0.294	0.16 [-0.29, 0.62]<br>$t_{(19)} = 0.74$<br>p = 0.469	0.43 [-5.59x10^{-4}, 0.85]<br>$t_{(19)} = 2.09$<br>p = 0.050	0.34 [-0.10, 0.78]<br>$t_{(19)} = 1.63$<br>p = 0.119
Glutamate* Outcome group[Stim]	**0.69 [0.27, 1.11]**<br>**$t_{(18)} = 3.45$**<br>**p = 0.003**	0.36 [-0.07, 0.79]<br>$t_{(18)} = 1.74$<br>p = 0.098	0.36 [-0.07, 0.79]<br>$t_{(18)} = 1.74$<br>p = 0.099	0.23 [-0.20, 0.66]<br>$t_{(18)} = 1.10$<br>p = 0.284	0.19 [-0.27, 0.64]<br>$t_{(18)} = 0.86$<br>p = 0.40	**0.43 [4.88x10^{-3}, 0.86]**<br>**$t_{(18)} = 2.12$**<br>**p = 0.048**	0.35 [-0.10, 0.79]<br>$t_{(18)} = 1.63$<br>p = 0.121

Glutamate* Outcome group[StimEver]	**0.70 [0.31, 1.09]** **$t_{(29)}$ = 3.65** **p =0.001**	0.34 [-0.06, 0.74] $t_{(29)}$ = 1.76 p = 0.089	0.32 [-0.08, 0.73] $t_{(29)}$ = 1.64 p = 0.112	0.21 [-0.19, 0.62] $t_{(29)}$ = 1.07 p = 0.295	0.10 [-0.33, 0.53] $t_{(29)}$ = 0.48 p = 0.631	**0.46 [0.05, 0.87]** **$t_{(29)}$ = 2.32** **p = 0.028**	0.42 [-4.6 x10^{-3}, 0.85] $t_{(29)}$ = 2.07 p = 0.048
Glutamate* Outcome group[Co-Dx]	**0.68 [0.23, 1.13]** **$t_{(18)}$ = 3.16** **p = 0.005**	0.30 [-0.16, 0.77] $t_{(18)}$ = 1.38 p = 0.184	0.30 [-0.16, 0.77] $t_{(18)}$ = 1.38 p = 0.186	0.14 [-0.33, 0.60] $t_{(18)}$ = 0.62 p = 0.542	0.11 [-0.38, 0.61] $t_{(18)}$ = 0.49 p = 0.629	**0.52 [0.07, 0.98]** **$t_{(18)}$ = 2.42** **p = 0.026**	0.28 [-0.19, 0.76] $t_{(18)}$ = 1.26 p = 0.224

Linear Mixed Model: $Network\_metric_{ij} \sim Glutamate_{ij} * outcome\ group_i + age_{jj} + sex_i + motion_{ij} + (motion_{ij})^2$
Random intercept: 1| Participant

Linear Mixed Model with two outcome groups only: Persistent ADHD vs. Never Affected (Reference group).
[IQ]: Model with full scale intellectual quotient (IQ) added as a covariate
[Stim]: Model with psychostimulant medication status at the time of MRI added as a covariate (participants were off psychostimulant the day of the scan, however, we kept tracked off ongoing psychostimulant therapy).
[StimEver]: Model with covariate "ever received psychostimulant treatment" added.
[Co-Dx]: Same model excluding 13 participants with comorbidities.

C) Primary analysis with the three outcome groups.

	Within DMN		DMN-dorsal attention network		DMN-central executive network		DMN-salience network		DMN-subcortical	
	Statistics	P- value	Statistics	P- value	Statistics	P- value	Statistics	P- value	Statistics	P- value
Glutamate	$F_{(1,31)}$=9.97	**0.004***	$F_{(1,31)}$=1.72	0.2	$F_{(1,31)}$=1.98	0.17	$F_{(1,31)}$=1.43	0.24	$F_{(1,31)}$=3.22	0.08
Outcome group	$F_{(2,100)}$=2.98	0.05	$F_{(2,100)}$=1.12	0.89	$F_{(2,100)}$=2.83	0.06	$F_{(2,100)}$=0.27	0.77	$F_{(2,100)}$=4.83	**0.01**
Sex	$F_{(1,100)}$=2.64	0.1	$F_{(1,100)}$=1.29	0.26	$F_{(1,100)}$=1.02	0.31	$F_{(1,100)}$=0.6	0.44	$F_{(1,100)}$=3.62	0.06
Motion	$F_{(1,31)}$=0.08	0.77	$F_{(1,31)}$=0.39	0.53	$F_{(1,31)}$=0.18	0.67	$F_{(1,31)}$=1.86	0.18	$F_{(1,31)}$=4.01	0.05
Motion^2	$F_{(1,31)}$=0.12	0.73	$F_{(1,31)}$=0.06	0.81	$F_{(1,31)}$=0.21	0.65	$F_{(1,31)}$=0.16	0.69	$F_{(1,31)}$=2.61	0.12
Age at scan	$F_{(1,31)}$=0.7	0.41	$F_{(1,31)}$=1.19	0.66	$F_{(1,31)}$=0.05	0.82	$F_{(1,31)}$=1.54	0.22	$F_{(1,31)}$=2.95	0.1
Glutamate* Outcome group	$F_{(2,31)}$=4.1	**0.045**	$F_{(2,31)}$=0.13	0.88	$F_{(2,31)}$=3.15	0.06	$F_{(2,31)}$=0.33	0.72	$F_{(2,31)}$=4.5	**0.02**

*Linear Mixed Model F-test with the three outcome groups. Bold indicate p<0.05, * p<0.01.*
Abbreviation: DMN, default node network.

		Persistent ADHD	Remitting ADHD	Never Affected
Within DMN	Estimate (SE)<br>[95% bootstrapped CI]	0.03 (0.01)<br>[0.01; 0.05]	0.02 (0.02)<br>[-0.01; 0.05]	-0.02 (0.01)<br>[-0.03; -0.01]
	Statistics –<br>*Reference Group: Never affected*	**$t_{(31)}$=2.49**<br>**p=0.018**	$t_{(31)}$=1.11<br>p=0.27	
	Statistics –<br>*Reference Group: Persistent ADHD*		$t_{(31)}$=-0.68<br>p=0.5	
DMN-Subcortical	Estimate (SE)<br>[95% bootstrapped CI]	0.03 (0.01)<br>[0.01;0.05]	0.02 (0.01)<br>[0; 0.05]	-0.01 (0.01)<br>[-0.02; 0]
	Statistics –<br>*Reference Group: Never affected*	**$t_{(31)}$=2.87**<br>**p=0.007***	$t_{(31)}$=1.57<br>p=0.13	
	Statistics –<br>*Reference Group: Persistent ADHD*		$t_{(31)}$=-0.49<br>p=0.63	

*Glutamate by outcome group interactions t-test from the linear mixed model. Bold indicate p<0.05, * p<0.01.*
CI, confidence interval; DMN, default node network; SE, standard error.

Figure 5. Glutamate and Default-Mode Network (DMN) Connectivity.

A) Primary Analysis

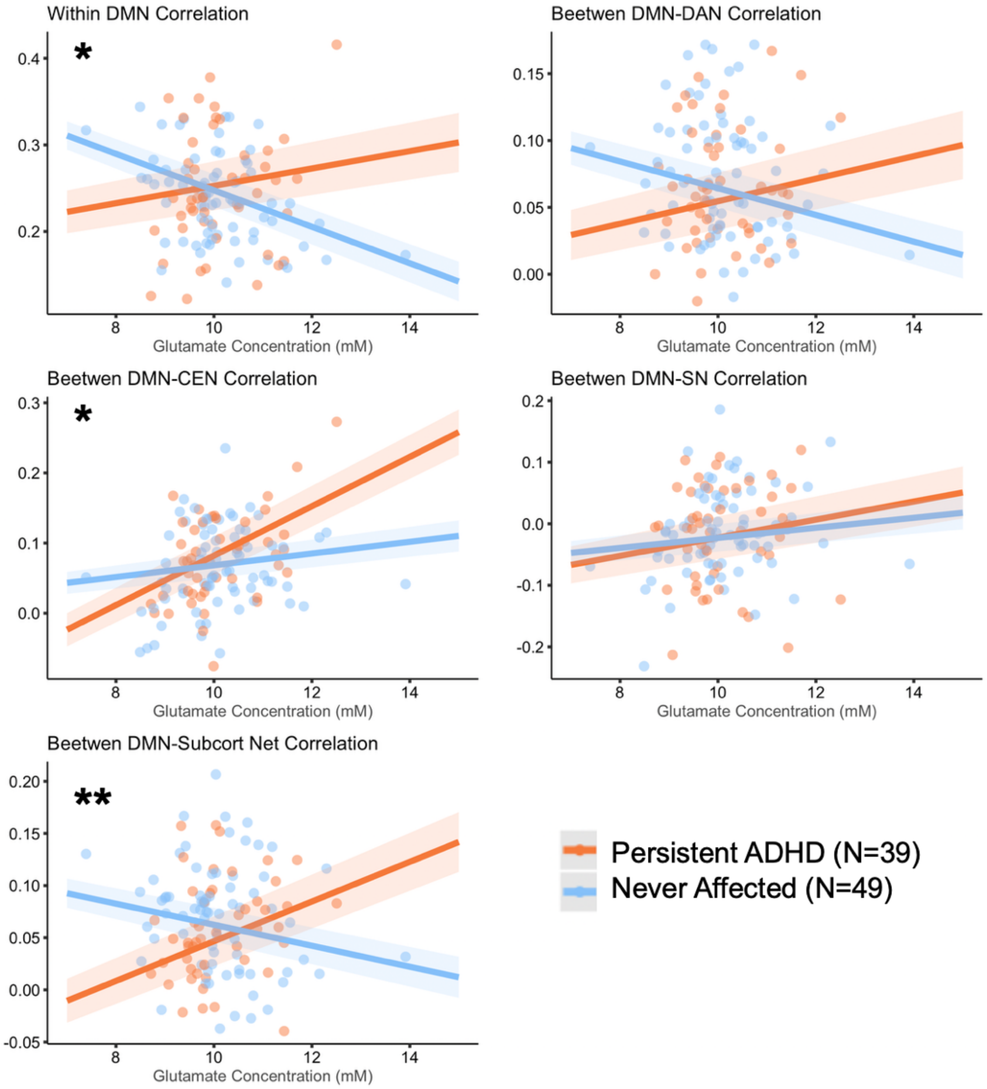

Points represent individual participant data and thick lines are the outcome group predicted fitted slope from the following mixed model: $Network\_metric_{ij} \sim Glutamate_{ij} * outcome\_group_i + age_{ij} + sex_i + motion_{ij} + (motion_{ij})^2$; with a random intercept term for subject (~1|ID). *indicate nominal significance and ** Bonferroni corrected significance. Slope estimates are reported in supplementary Table 8.A.

Abbreviations: CEN: Central Executive Network; DAN: Dorsal Attention Network; DMN, default node network; SN: Salience Network; Subcort Net: Subcortical Network.

B) Post-Hoc Analysis: Investigating the subcortical divisions.

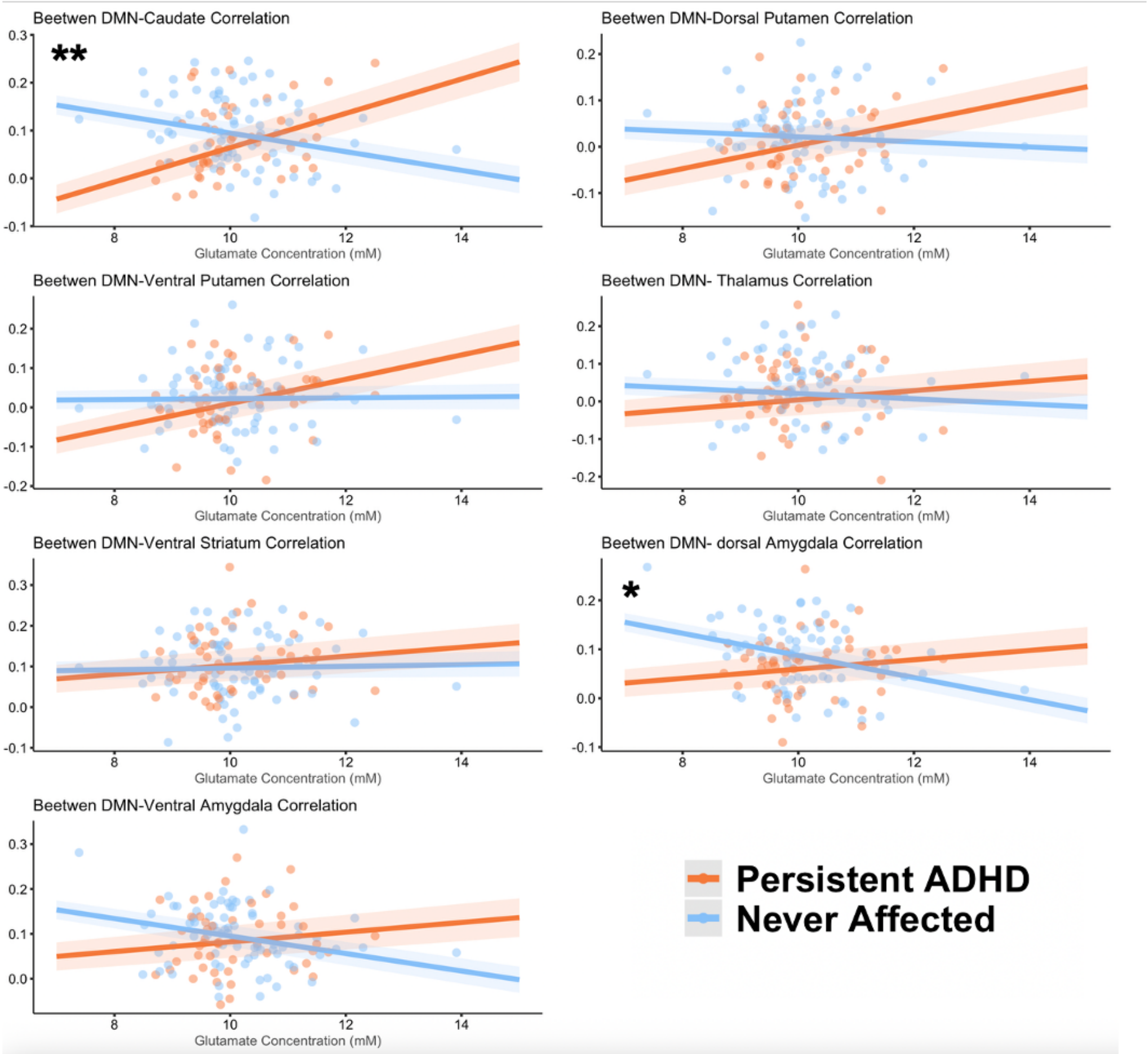

Points represent individual participant data and thick lines are the outcome group predicted fitted slope from the following mixed model: $Network\_metric_{ij} \sim Glutamate_{ij} * outcome\_group_i + age_{ij} + sex_i + motion_{ij} + (motion_{ij})^2$; with a random intercept term for subject (~1|ID). *indicate significance. Slope estimates are reported in supplementary Table 8.B.

Abbreviation: DMN, default node network.

References

1. Lin A, Andronesi O, Bogner W, Choi IY, Coello E, Cudalbu C*, et al*. Minimum Reporting Standards for in vivo Magnetic Resonance Spectroscopy (MRSinMRS): Experts' consensus recommendations. *NMR Biomed*, 2021. **34**(5): p. e4484.
2. Zhang Y and Shen J. Simultaneous quantification of glutamate and glutamine by J-modulated spectroscopy at 3 Tesla. *Magn Reson Med*, 2016. **76**(3): p. 725-32.
3. Zhang Y, An L, and Shen J. Fast computation of full density matrix of multispin systems for spatially localized in vivo magnetic resonance spectroscopy. *Med Phys*, 2017. **44**(8): p. 4169-4178.
4. Fischl B, Salat DH, Busa E, Albert M, Dieterich M, Haselgrove C*, et al*. Whole brain segmentation: automated labeling of neuroanatomical structures in the human brain. *Neuron*, 2002. **33**(3): p. 341-55.
5. Ernst T, Kreis R, and Ross BD. Absolute quantitation of water and metabolites in human brain. *J. Magn. Reson.*, 1993. **102**: p. 1-8.
6. Jenkinson M, Bannister P, Brady M, and Smith S. Improved optimization for the robust and accurate linear registration and motion correction of brain images. *Neuroimage*, 2002. **17**(2): p. 825-41.
